# Can X-ray constrained Hartree–Fock wavefunctions retrieve electron correlation?

**DOI:** 10.1107/S2052252516019217

**Published:** 2017-01-10

**Authors:** Alessandro Genoni, Leonardo H. R. Dos Santos, Benjamin Meyer, Piero Macchi

**Affiliations:** a CNRS, Laboratoire SRSMC, UMR 7565, Boulevard des Aiguillettes, BP 70239, Vandoeuvre-lès-Nancy, F-54506, France; b Université de Lorraine, Laboratoire SRSMC, UMR 7565, Boulevard des Aiguillettes, BP 70239, Vandoeuvre-lès-Nancy, F-54506, France; c Department of Chemistry and Biochemistry, University of Bern, Freiestrasse 3, Bern 3012, Switzerland

**Keywords:** electron correlation, electron density, X-ray diffraction, X-ray constrained wavefunctions, constrained Hartree–Fock wavefunctions, density functional theory

## Abstract

The X-ray constrained wavefunction (XC-WF) method proposed by Jayatilaka [Jayatilaka & Grimwood (2001) ▸, *Acta Cryst.* A**57**, 76–86] has attracted much attention because it represents a possible third way of theoretically studying the electronic structure of atoms and molecules, combining features of the more popular wavefunction- and DFT-based approaches. In its original formulation, the XC-WF technique extracts statistically plausible wavefunctions from experimental X-ray diffraction data of molecular crystals. A weight is used to constrain the pure Hartree–Fock solution to the observed X-ray structure factors. Despite the wavefunction being a single Slater determinant, it is generally assumed that its flexibility could guarantee the capture, better than any other experimental model, of electron correlation effects, absent in the Hartree–Fock Hamiltonian but present in the structure factors measured experimentally. However, although the approach has been known for long time, careful testing of this fundamental hypothesis is still missing. Since a formal demonstration is impossible, the validation can only be done heuristically and, to accomplish this task, X-ray constrained Hartree–Fock calculations have been performed using structure factor amplitudes computed at a very high correlation level (coupled cluster) for selected molecules in isolation, in order to avoid the perturbations due to intermolecular interactions. The results show that a single-determinant XC-WF is able to capture the electron correlation effects only partially. The largest amount of electron correlation is extracted when: (i) a large external weight is used (much larger than what has normally been used in XC-WF calculations using experimental data); and (ii) the high-order reflections, which carry less information on the electron correlation, are down-weighted (or even excluded), otherwise they would bias the fitting towards the unconstrained Hartree–Fock wavefunction.

## Abstract 2

In this study, the X-ray constrained wavefunction approach is carefully investigated in order to assess its ability to capture the effect of electron correlation on electron density. Electron distributions obtained from highly correlated molecular wavefunctions are the benchmarks and their Fourier transforms are used to simulate X-ray intensities for the constrained wavefunction calculations.

## 1. Introduction   

As is well known, X-ray scattering is the Fourier image of the dynamic electron-density distribution. It is now well established that, by fitting suitable multipole models or through maximum likelihood estimations, one can reconstruct accurate electron-density maps from the X-ray diffraction of a crystal. Furthermore, the technical developments that have occurred over the past few years constantly encourage the ever deeper exploration of the information contained in X-ray diffraction amplitudes. There are three main levels of detail that one may observe: (i) the hybridization of the atomic orbitals due to chemical bonding; (ii) the electron polarization caused by inter- or intramolecular electrostatic interactions; and (iii) the tiny electron-density redistribution, which reflects the instantaneous electron–electron repulsions (electron correlation). Details of the first kind, *i.e.* deformations from the spherical distributions around the atoms, are due to the formation of covalent bonds or to the localization of lone pairs. They can be easily observed and they were the first to be visualized in the early experiments of the 1960s (Coppens, 1967 ▸). Intra- or intermolecular electrostatic interactions induce electron-density polarizations, which are obviously proportional to the strength of the field. In strong hydrogen bonds, polarization of the lone pairs of acceptor atoms has been visualized since the 1980s (Stevens & Coppens, 1980 ▸). More recent studies have demonstrated that even much weaker interactions (like agostic bonds) leave clearly visible fingerprints in the electron-density distribution (Scherer *et al.*, 2015 ▸). The third level of electron-density deformation features is, in contrast, much subtler, because it is much more difficult to visualize and quantify the deformations produced by electron correlation. Detecting relativistic effects in single-crystal X-ray diffraction experiments is equally difficult, as recently discussed by Bučinský *et al.* (2016 ▸) and by Batke & Eickerling (2016 ▸).

Correct treatment of the electron correlation is crucial for a proper theoretical description of molecules and solids (Szabo & Ostlund, 1996 ▸; Helgaker *et al.*, 2000 ▸), in particular for obtaining reliable and detailed pictures of the chemical bonding, the electronic transitions and the dynamics of a system. This continuously fosters new theoretical strategies aiming to include as much electron correlation as possible. In this context, a role of paramount importance is obviously played by the well established post-Hartree–Fock *ab initio* techniques (Szabo & Ostlund, 1996 ▸; Helgaker *et al.*, 2000 ▸), *e.g.* configuration interaction, coupled cluster and many-body perturbation methods. These strategies are based on a multi-determinant wavefunction *Ansatz* and they enable the systematic improvement of the description of many-electron systems, despite their generally large computational cost. Of course, the preferred methods are those maximizing the marginal utility of these expensive theoretical calculations. A completely different approach is represented by the density functional theory (DFT; Parr & Yang, 1989 ▸; Perdew *et al.*, 2009 ▸; Jones, 2015 ▸) which, relying on the Hohenberg and Kohn theorem (Hohenberg & Kohn, 1964 ▸), is in principle exact and has the great computational advantage of using only the three-dimensional electron density as a basic physical entity, instead of the multi-variable wavefunction. Nevertheless, the exact functional relationship between the ground-state electron density and the ground-state energy of an electronic system remains unknown. Therefore, mainly exploiting the Kohn–Sham approach (Kohn & Sham, 1965 ▸), all the currently known DFT-based strategies allow only approximate treatments of the electron correlation using exchange-correlation functionals. These are ranked in classes of increasing complexity (Riley *et al.*, 2007 ▸), as for example in the ‘Jacob’s ladder’ proposed by Perdew & Schmidt (2001 ▸). However, unlike the post-Hartree–Fock methods, moving upwards from one rung of the ladder to the next does not always guarantee a systematic improvement in the calculations.

Although not yet commonly adopted by the theoretical chemistry community, a third way of investigating many-electron systems is the X-ray constrained wavefunction (XC-WF) approach, originally proposed by Jayatilaka and co-workers (Jayatilaka, 1998 ▸, 2012 ▸; Jayatilaka & Grimwood, 2001 ▸; Grimwood & Jayatilaka, 2001 ▸; Bytheway, Grimwood, Figgis *et al.*, 2002 ▸; Bytheway, Grimwood & Jayatilaka, 2002 ▸; Grimwood *et al.*, 2003 ▸). This technique is one of the most successful developments of the pioneering strategies introduced by Clinton and Massa since the 1960s (Clinton *et al.*, 1969 ▸; Clinton & Massa, 1972 ▸; Clinton *et al.*, 1973 ▸; Frishberg & Massa, 1981 ▸) and it allows the extraction of plausible model wavefunctions from experimental X-ray diffraction data. The XC-WF method can be considered as a way of merging wavefunction and DFT methods. In principle, this strategy should intrinsically be able to introduce electron correlation effects because it uses real observations. This could automatically lead to the definition of ‘correlation density’, which can be regarded as the difference between charge distributions corresponding to X-ray constrained and unconstrained Hartree–Fock wavefunctions. This possibility was one of the initial motivations of Jayatilaka’s work, but two main drawbacks undermine the ease of this interpretation: (i) an X-ray constrained molecular wavefunction is computed, on the one hand, exploiting the Hartree–Fock Hamiltonian for the isolated molecule, which neglects intermolecular fields, and, on the other hand, imposing a constraint on the X-ray intensities, which include the crystal field effects; and (ii) the experimental errors that affect the measurements and propagate in the wavefunction coefficients.

Apart from these two caveats, so far no accurate analysis has ever been attempted to demonstrate whether the X-ray constrained wavefunctions are indeed able to incorporate the effects of electron correlation. Therefore, the main goal of this paper is to investigate whether, and to what extent, the Jayatilaka approach and all its later developments (Hudák *et al.*, 2010 ▸; Genoni, 2013 ▸ *a*,*b* ▸; Dos Santos *et al.*, 2014 ▸; Genoni & Meyer, 2016 ▸) are actually intrinsically able to capture the effect of electron correlation on electron density. This is crucial, not only to assess further the capabilities of the X-ray constrained wavefunction methods, but also to open up unprecedented perspectives in the search for new density functionals, since, to the best of our knowledge, experimental (or theoretical) X-ray structure factors have not been exploited for this purpose.

**Figure 1:**
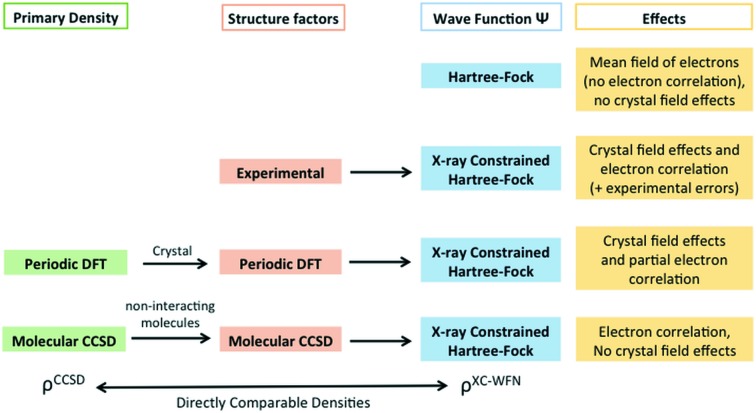
Possible X-ray constrained wavefunctions obtainable using different types of structure factors as constraints. The effects potentially captured by these X-ray constrained wavefunctions are also indicated.

Because of the above-mentioned pitfalls, we identified only one way to answer unequivocally the question in the title of this article. The strategy consists of testing whether, by the reciprocal-space constraint, an intrinsically uncorrelated single Slater determinant wavefunction is able to reproduce the electron distribution of a highly correlated wavefunction for an isolated molecule. This procedure does not make use of experimental data, where the information is actually convoluted with experimental errors and crystal field perturbations and lacks a suitable reference. At the same time, it does not use theoretical structure factors obtained from *ab initio* periodic calculations, because the effects of electron correlation and intermolecular interactions would be entangled. All these possibilities are synthetically schematized in Fig. 1 ▸.

The paper is organized as follows. First, we will briefly review the theory of the Jayatilaka approach and we will dedicate a section to the computational details of our investigation. We will then show and comment on the obtained results, and finally we will draw general conclusions.

## 2. Theoretical background   

For the sake of completeness, we remind the reader that the XC-WF strategy assumes that it is working with an effective molecular crystal constituted by non-interacting units described by electronic wavefunctions that are formally identical and mutually related through the crystal symmetry operations. Moreover, the assumption that each non-interacting unit can be associated with a symmetry-unique portion of the crystal unit cell enables the expression of the unit-cell electron density as a sum of *N_m_* crystal-unit electron distributions ρ_*k*_(**r**), which can be simply obtained from the reference distribution ρ_0_(**r**) through the unit-cell symmetry operations {**R**_*k*_, **r**_*k*_} 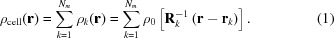
Equation (1) is strictly exact if ρ_0_(**r**) is an exact partition of the total electron density of the unit cell. To guarantee this, in Jayatilaka’s approach ρ_0_(**r**) is the electron density associated with the single Slater determinant that not only variationally minimizes the electronic energy of the reference unit, but also reproduces a set of observed structure factor amplitudes 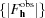, measured experimentally or calculated theoretically. In other words, an external constraint ensures that the global electron density of the fictitious non-interacting crystal is identical to the electron distribution of the corresponding real interacting system (Jayatilaka & Grimwood, 2001 ▸). Therefore, the method becomes equivalent to finding the molecular orbitals (MOs) of the Slater determinant that minimize the following functional: 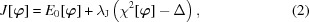
where *E*_0_ is the energy associated with the Slater determinant of the reference crystal unit, λ_J_ is an external adjustable parameter that is varied during the calculations and which represents the strength of the external constraint, χ^2^ is the measure of statistical agreement between the calculated and experimentally (or theoretically) collected structure factor amplitudes, Δ is the desired agreement between those quantities (typically fixed to 1.0 in the case of experimental data) and 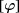 stresses the functional dependence on the occupied MOs. In particular, χ^2^ is expressed as 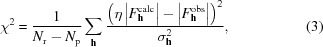
with *N*_r_ the number of collected X-ray diffraction data, *N*_p_ the number of adjustable parameters (in this case only the external multiplier λ_J_), **h** the triad of Miller indices labelling the reflection, σ_**h**_ the standard uncertainty corresponding to each observed structure factor amplitude 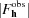 and η a scale factor that, in the case of experimental constraints, is properly determined in order to minimize χ^2^, whereas in the case of theoretical constraints it is simply set equal to 1.0 (Genoni, 2013*b* ▸).

Now, exploiting the definition of the structure factor operator 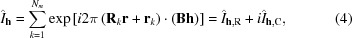
where **B** is the reciprocal-lattice matrix and 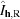 and 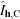 are both hermitian operators, it is easy to show that finding the MOs that minimize the functional of equation (2) is equivalent to solving the following modified Hartree–Fock equation: 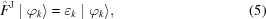
where the Fock–Jayatilaka operator 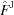 is given by 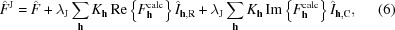
with 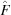 the usual Fock operator and with the multiplicative constant *K*_**h**_ expressed as 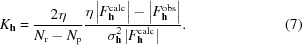



## 3. Computational details   

### 3.1. Computational strategy   

To conduct our investigations we considered six small/medium-sized systems, namely the two very simple diatomic molecules N_2_ (nitrogen molecule) and CN^−^ (cyanide anion) and the polyatomic molecules water, urea, benzene and glycine. For each of them we performed both traditional quantum chemistry calculations and X-ray constrained wavefunction computations, described below.

#### 3.1.1. Gas-phase molecular orbital calculations   

The geometries of the different molecules were optimized at the CCSD (coupled cluster with single and double excitations; Čížek, 1966 ▸; Purvis & Bartlett, 1982 ▸) level with the 6-311++G(2d,2p) basis set. Afterwards, using the same set of basis functions and the obtained minimum structure, traditional single-point calculations were performed at the RHF (restricted Hartree–Fock), CISD (configuration interaction with single and double excitations; Shavitt, 1977 ▸) and DFT levels. In particular, for the DFT calculations we adopted the BLYP (Becke, 1988 ▸; Lee *et al.*, 1998 ▸), B3LYP (Becke, 1988 ▸; Lee *et al.*, 1998 ▸; Stephens *et al.*, 1994 ▸; Hertwig & Koch, 1997 ▸), VSXC (Van Voorhis & Scuseria, 1998 ▸) and B1B95 (Becke, 1988 ▸; Becke, 1996 ▸) functionals, which are GGA (generalized gradient approximation), hybrid-GGA, meta-GGA and hybrid-meta-GGA functionals, respectively. All these traditional quantum chemistry computations were carried out using the *GAUSSIAN09* package (Frisch *et al.*, 2009 ▸).

#### 3.1.2. Structure factor computation   

After determination of the CCSD/6-311++G(2d,2p) molecular electron densities, for each system we computed theoretical X-ray structure factor amplitudes as analytic Fourier transforms up to a resolution sinθ/λ = 2.0 Å^−1^. Reciprocal space was sampled with different unit-cell sizes, either cubic with a large unit-cell edge or adapted to the molecular volume and shape. The unit-cell volume played only a negligible role, therefore for the sake of homogeneity all results presented in this paper refer to a constant type of unit cell (*a* = *b* = *c* = 10.0 Å), all tested to be sufficiently large for the molecules under examination.

**Figure 2:**
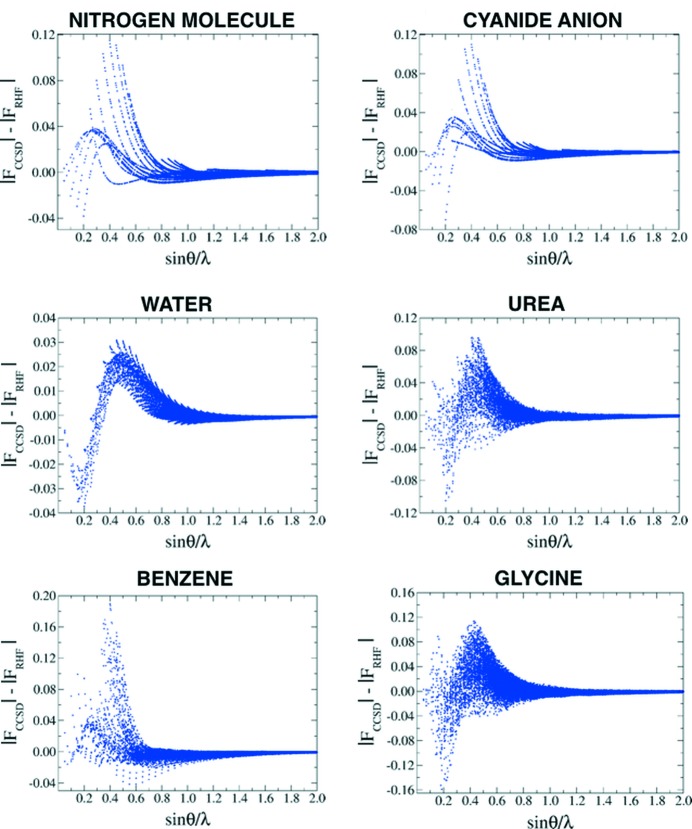
Differences between the CCSD and the RHF structure factor amplitudes as a function of the resolution sinθ/λ for the six different molecules taken into account.

Using a gas-phase molecular electron density, we avoided any intermolecular interaction in the estimation of the structure factors and, using a large unit cell, we also avoided any artefact due to the superposition of vicinal unit-cell densities. The same procedure was used for the calculation of the theoretical structure factor amplitudes at the RHF/6-311++G(2d,2p) level. The CCSD and RHF reflections were compared afterwards. In Fig. 2 ▸ it is easy to observe that, for most of the high-angle reflections, the difference between the CCSD and RHF values is very small. This is not surprising, because electron correlation is expected to modify the contracted (core) electron density only as an indirect effect of the chemical bonding (Gatti *et al.*, 1988 ▸; Boyd & Wang, 1989 ▸). On the contrary, the largest discrepancies occur at low angles.

#### 3.1.3. X-ray constrained Hartree–Fock calculations   

The *ab initio* CCSD structure factor amplitudes were then exploited to perform X-ray constrained restricted Hartree–Fock (XC-RHF) calculations (Jayatilaka & Grimwood, 2003 ▸) using the same 6-311++G(2d,2p) basis set. In particular, all the XC-RHF computations were carried out (i) considering a pseudo-crystal with space group *P*1 and with the same pseudo-cubic unit cell adopted for the computation of the theoretical reflections, and (ii) varying the adjustable parameter λ_J_ from 0 to 10.0 with a step of 0.5. Furthermore, as well as performing calculations with only the complete set of reflections (sinθ/λ ≤ 2.0 Å^−1^), six further subsets of decreasing maximum resolution were also selected (sinθ/λ ≤ 1.5, 1.2, 0.9, 0.7, 0.5, 0.25 Å^−1^) in our X-ray constrained computations. Since in all the considered cases only purely theoretical structure factor amplitudes were used as constraints, the standard uncertainties σ_**h**_ and the scale factor η [see equation (3)] were set equal to 1.0. Furthermore, treatment of the thermal motion has been completely neglected (*i.e.* anisotropic displacement parameters set to 0.0). All the XC-RHF calculations were carried out using the free software *TONTO* (Jayatilaka & Grimwood, 2003 ▸).

### 3.2. Comparison of the electron densities   

As already mentioned in the *Introduction*, our main goal is to determine whether and to what extent the X-ray constrained wavefunction approach is able to capture the effects of the electron correlation on electron density. To accomplish this task, we have mainly compared the obtained electron densities by considering two different benchmarks: (i) the RHF charge density, corresponding to a totally uncorrelated electron distribution; and (ii) the CCSD electron density, *i.e.* the correlated charge distribution from which we calculated the structure factors. In particular, to analyse the differences between the various electron densities, we have used several indicators that are briefly described here.

#### 3.2.1. Topological agreement index   

This index, TI, is calculated from the electron densities at the different topological bond critical points (**r**_b_) and it is defined as 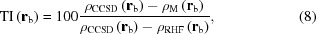
where ρ_CCSD_(**r**_b_) is the electron density at the CCSD level of theory, ρ_RHF_(**r**_b_) is the unconstrained and uncorrelated Hartree–Fock electron-density distribution and ρ_M_(**r**_b_) is the charge density to be compared, namely a charge density obtained from one of the considered ‘correlated methods’ (*i.e.* obtained from an XC-WF, a DFT or another post-Hartree–Fock calculation). Of course, for complete similarity with the CCSD and RHF benchmark values, the topological agreement index is equal to 0 or 100, respectively. A similar index could also be defined for other functions (for example the Laplacian, the electron-density gradient, the electrostatic potential *etc*.) evaluated at the bond or at other critical points. Here, however, we report results only for ρ(**r**_b_).

#### 3.2.2. Real-space *R* (RSR) value   

The RSR value is calculated on a grid of *n*_p_ points as (Jones *et al.*, 1991 ▸)
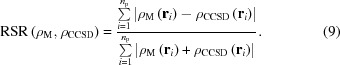
In this case, only the CCSD electron density has been used as a reference and complete similarity between the CCSD and the model M charge distribution under examination corresponds to RSR = 0.

#### 3.2.3. Euclidean Carbó distance   

The Euclidean distance *d_IJ_* between two molecular electron distributions ρ_*I*_(**r**) and ρ_*J*_(**r**) was defined by Carbó and co-workers (Carbó *et al.*, 1980 ▸; Carbó & Calabuig, 1992 ▸) as 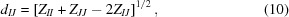
where the overlap-like similarity *Z_IJ_* is given by 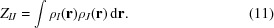
Of course, complete similarity occurs when *d_IJ_* is equal to zero, while smaller similarities correspond to larger values of the index.

#### 3.2.4. Root-mean-square deviation and mean absolute deviation   

For the sake of completeness, the more traditional root-mean-square deviation (RMSD) and mean absolute deviation (MAD) between two electron densities were also considered. They are defined, respectively, as 
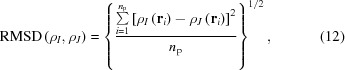
and 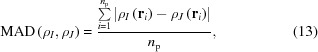
with *n*_p_ the number of points on the electron-density grids.

#### 3.2.5. Attachment and detachment densities   

Finally, another indicator of the amount of electron correlation captured by means of the XC-WF calculations is provided by the detachment and attachment densities (Head-Gordon *et al.*, 1995 ▸). The detachment density is originally defined as that part of the ground-state electron distribution that is removed and rearranged into the attachment electron density after an electronic transition. In our study, for all the examined molecules, the unconstrained RHF wavefunctions always represented our ‘pseudo-ground states’ (starting states), while the correlated wavefunctions (*i.e.* the CCSD, CISD and XC-RHF wavefunctions) were considered as ‘pseudo-excited states’ (final states).

## 4. Results and discussion   

**Figure 3:**
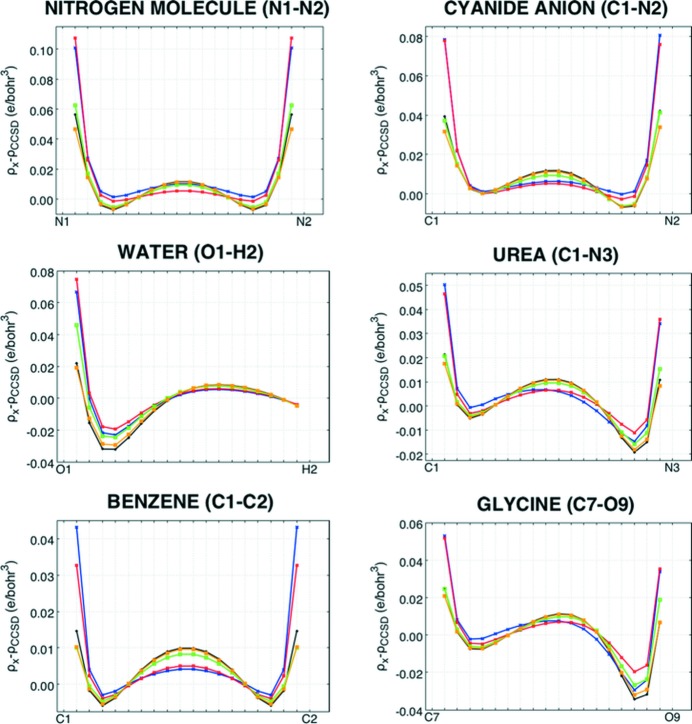
Comparison between the CCSD electron densities and the RHF (black), XC-WF/0.5 (blue), XC-WF/0.7 (red), XC-WF/1.2 (green) and XC-WF/2.0 (orange) charge distributions along some selected chemical bonds of the six investigated molecules.

In agreement with previous studies (Gatti *et al.*, 1988 ▸; Boyd & Wang, 1989 ▸), the effect of the electron correlation on ρ(**r**) is tiny and therefore difficult to capture, especially for methods based on fitting procedures like the X-ray constrained wavefunction or the more traditional multipolar expansions. Electron correlation generally reduces the electron density in the bonding regions in favour of the core ones. As a matter of fact, all the correlated methods (*i.e.* the CCSD, CISD, DFT methods) as well as the X-ray constrained Hartree–Fock strategy generally return smaller ρ(**r**_b_) values than the uncorrelated RHF technique for all the bonds that we have investigated (see, for instance, the topological agreement index in Tables 1 ▸–3 ▸ ▸ for the N—N, C—N and O—H bond critical points of the nitrogen, cyanide and water molecules, and in Tables S1–S13 of the supporting information for the bond critical points of the other investigated systems). This agrees with the results of previous investigations (Bader & Chandra, 1968 ▸; Smith, 1977 ▸; Moszyński & Szalewicz, 1987 ▸). The trend is also evident when analysing ρ(**r**) along the covalent bonds of the investigated molecules (see Fig. 3 ▸, and Fig. S1 in the supporting information). In fact, the RHF electron density is systematically larger than the CCSD one in the middle of all the bonds.

Obviously, any X-ray constrained RHF wavefunction that at least partially includes the effects of electron correlation should reduce the difference between the CCSD and the uncorrelated RHF electron-density distributions. The behaviour of partially correlated wavefunctions is quite inhomogeneous and, therefore, the response at one point only (like the bond critical point) or along a line (like the bond path) may not be sufficiently representative. Therefore, for a more comprehensive overview, the whole set of indicators described in Section 3.2 is necessary. These indices allowed us to assess the global performances of the X-ray constrained wavefunctions and, together with the other more local indicators mentioned above, enabled us to analyse the following features:

(i) The overall similarity between the charge distributions, by means of the real-space *R* (RSR) value, the Carbó distance and the RMSD and MAD indexes;

(ii) The displacement of electron density due to electron correlation, visualized by electron-density differences along representative chemical bonds, as well as by difference density maps and attachment and detachment densities;

(iii) The properties of the electron densities at some special points, like the bond critical points (BCPs) of the molecules, that are often used to infer the nature of chemical bonds.

**Figure 4:**
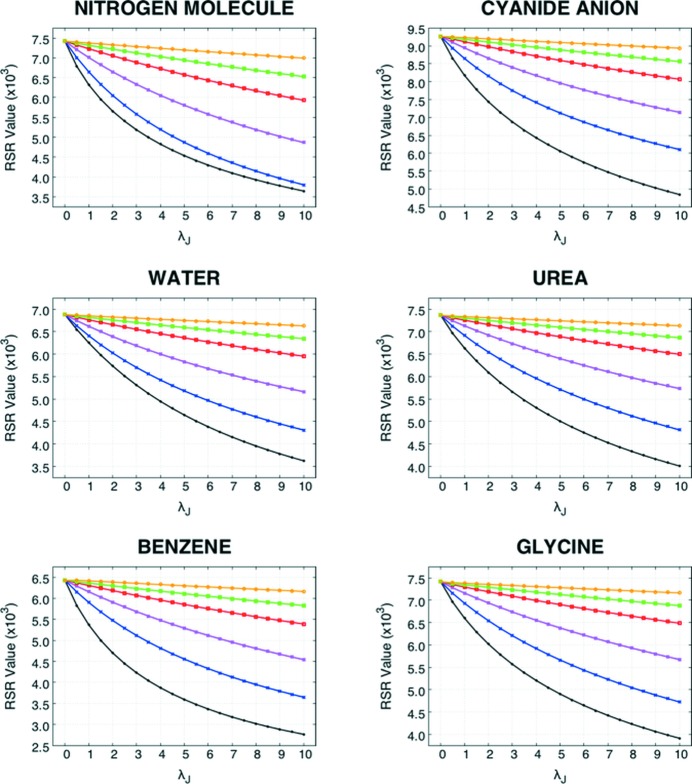
Values of the RSR similarity indexes between the CCSD electron densities and the XC-WF charge distributions as a function of the external multiplier λ_J_ for all the examined molecules. The yellow, green, red, magenta, blue and black curves correspond to XC-WF calculations performed with structure factor amplitudes up to a resolution of 2.0, 1.5, 1.2, 0.9, 0.7 and 0.5 Å^−1^, respectively.

**Figure 5:**
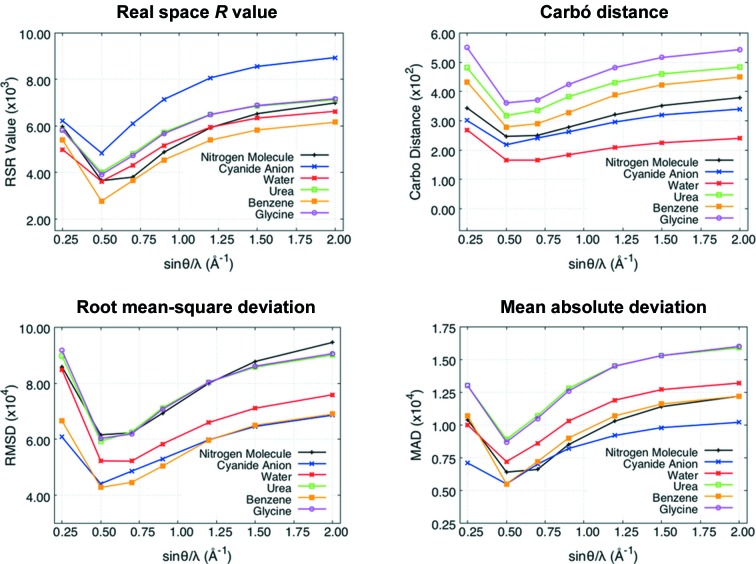
Values of the similarity indexes between the CCSD electron densities and the XC-WF charge distributions (λ_J_ = 10.0) as a function of the resolution sinθ/λ for all the molecules taken into account.

**Figure 6:**
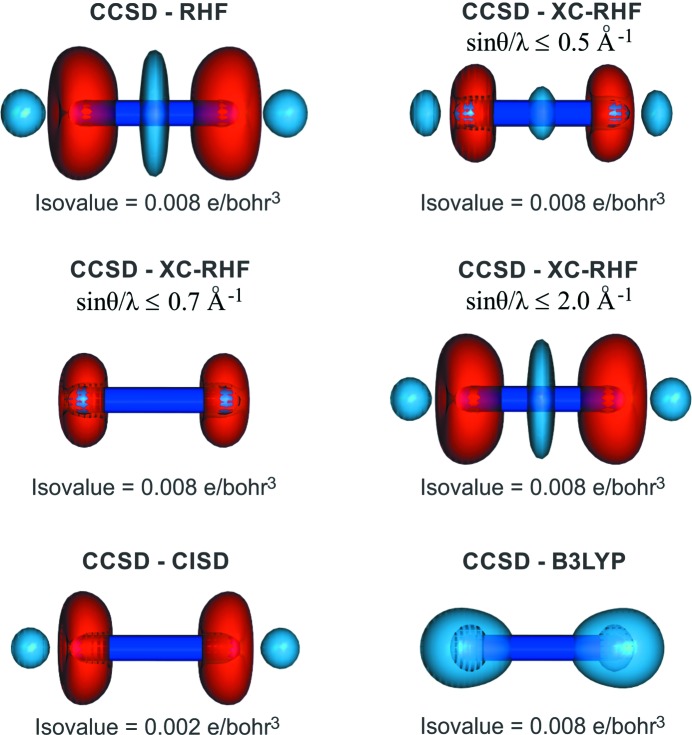
Representative isosurfaces of the difference density maps for the nitrogen molecule, using the CCSD charge distribution as reference. The chosen isovalue is always 0.008 e bohr^−3^, except for the CCSD–CISD difference density, for which the isovalue has been set equal to 0.002 e bohr^−3^. Positive and negative isosurfaces are depicted in red and blue, respectively.

**Figure 7:**
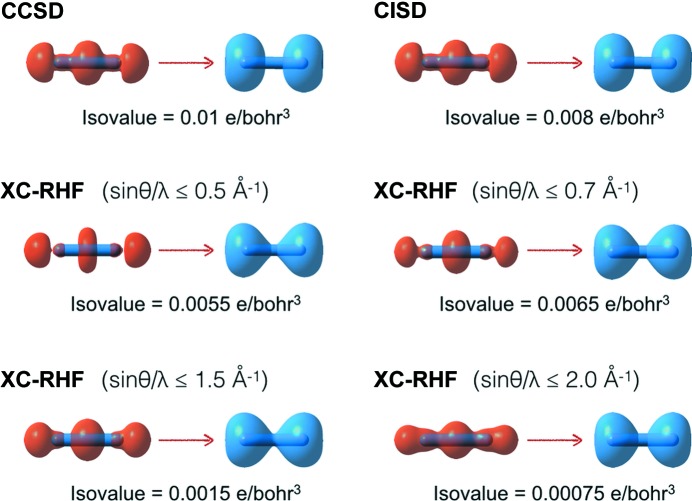
Representative isosurfaces of the detachment and attachment densities (in orange and blue, respectively) of the nitrogen molecule relative to electronic rearrangements with respect to the reference RHF charge distribution when different correlated wavefunctions are taken into account.

**Figure 8:**
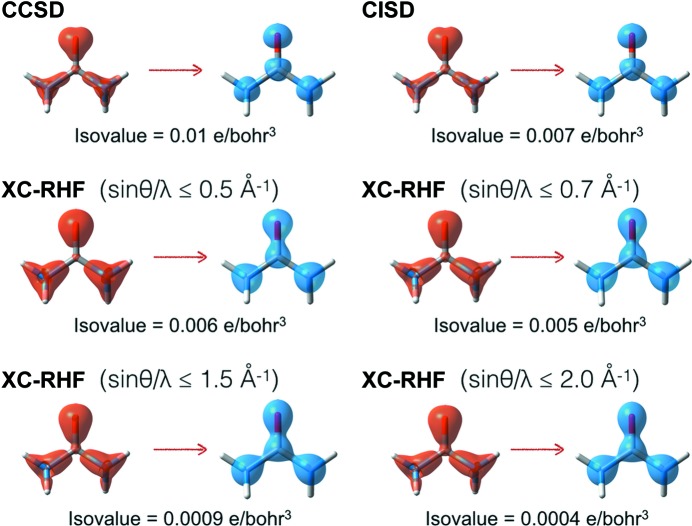
Representative isosurfaces of the detachment and attachment densities (in orange and blue, respectively) of the urea molecule relative to electronic rearrangements with respect to the reference RHF charge distribution when different correlated wavefunctions are taken into account.

From Figs. 3–8, we can observe some general trends, which are valid for all the investigated systems.

Due to the dual nature of the functional in equation (2), an X-ray constrained wavefunction cannot deviate too much from the RHF one if λ_J_ is small. From Fig. 4 ▸ (and from Figs. S2–S4 in the supporting information), it is obvious that, given a particular resolution limit, the correlation effects are significantly included only if the external weight λ_J_ is sufficiently large. In fact, a large λ_J_ implies a more precise fitting of the experimental data and a Jayatilaka functional [see equation (2)] dominated by a term that may effectively account for the electron correlation. In traditional XC-WF calculations, a λ_J_ ≤ 1 is normally adopted, which implies that the X-ray constrained electron densities remain too similar to those associated with the unconstrained and uncorrelated RHF wavefunctions.

As shown in Fig. 5 ▸, the amount of electron correlation effects captured by the XC-WFs is also strongly resolution dependent (see also Fig. S5 of the supporting information). For a given weight λ_J_, the largest recoveries generally occur for calculations only including reflections up to sinθ/λ = 0.5–0.7 Å^−1^. It is likely that this range may change for molecules containing atoms on the third row of the periodic table or below, because of the different radial distributions of the core and valence densities of those atoms. In order to understand this behaviour, we have to consider that the correlation effects in the valence region of second-row elements affect the structure factors up to a resolution of *ca* sinθ/λ = 0.7 Å^−1^, whereas the correlation effects in the core region affect all reflections up to infinite resolution, well above sinθ/λ = 2.0 Å^−1^. However, the electron redistribution due to electron correlation is sufficient to produce an oscillation of the structure factor changes, which is clearly visible in Fig. 2 ▸. Moreover, the higher resolution reflections are individually less perturbed. A refinement including reflections up to sinθ/λ = 0.7 Å^−1^ comprehensively describes the effects of electron correlation on the valence density, whereas at lower resolution (*e.g.* 0.25 Å^−1^) this fitting is incomplete. On the contrary, at higher resolution, since only part of the core electron density is fitted, the oscillating nature of the perturbation is such that the XC-WF charge distributions basically coincide with the RHF one (see Fig. 2 ▸), thus losing the correctness of the medium-resolution fit. Only at an extremely high resolution, *i.e.* well above reasonably affordable limits, may the electron correlation effects be fully recovered.

For this reason, the differences between electron densities corresponding to XC-WF and RHF or CCSD wavefunctions are rather heterogeneously distributed. In the bonding region, the electron density of a medium-resolution XC-WF is closer to that associated with the correlated CCSD wavefunction, whereas the agreement is much poorer in the vicinity of the nuclei. In Fig. 3 ▸, this is particularly evident for the representative bonds in the cyanide, urea and benzene molecules. As explained above, high-resolution XC-WFs behave very similarly to the RHF ones. The associated electron densities deviate more substantially from the corresponding CCSD charge distributions, especially in the bonding region. This picture also emerges when analysing the difference densities for the investigated systems. In Fig. 6 ▸, we report the representative isosurfaces of the difference density maps computed for the nitrogen molecule using the CCSD electron distribution as a reference. At high resolution, the CCSD/XC-WF difference map is practically identical to the CCSD/RHF one, while at medium resolution (sinθ/λ ≤ 0.7 Å^−1^) the discrepancies in the bonding region disappear and those in the vicinity of the nuclei remain. This figure also confirms what was already observed in Fig. 5 ▸, which indicated that the largest recoveries of electron correlation generally occur when only low- to medium-angle reflections are considered in the calculations. As expected, the electron density resulting from the CISD wavefunction significantly approaches the CCSD charge distribution (for example in Fig. 6 ▸, consider that the isovalue necessary to visualize the CCSD–CISD difference density maps is much smaller than that used to plot the other isosurfaces). On the other hand, the behaviour of the density functional methods is less systematic. The trends observed in Fig. 6 ▸ are common to all the examined systems and, for the sake of proof, in the supporting information we have reported analogous difference density maps for the larger urea mol­ecule (see Fig. S6).

All the observations reported here are quite significant and in part surprising. It is worth stressing that large values of λ_J_ are necessary in order to obtain XC-WFs whose associated electron densities fit, at least partially, the effects missing in the Hamiltonian part of the functional given in equation (2) but which are present only in the constraint to the experimental data. Furthermore, it is surprising that a constraint to a set of reflections up to high resolution reduces the ability to capture these effects. As explained above, a full electron-density fit (an ideal XC-WF procedure with λ_J_ → ∞ and sinθ/λ → ∞) should converge to the correct electron density. In fact, the trends of the RSR similarity index as a function of the weight parameter λ_J_ (see Fig. 4 ▸) indicate that the high-resolution XC-WF slowly but constantly deviates from the RHF solution as λ_J_ increases. For relatively small λ_J_ values, a medium/low-resolution XC-WF deviates more rapidly from the Hartree–Fock model than does a high-resolution XC-WF, which shows a much slower convergence (almost perfectly linear up to λ_J_ = 10.0).

The asymptotic behaviour is actually difficult to assess, given that, for very large values of λ_J_, the convergence of the self-consistent field cycles can be difficult or even impossible. Anyway, for all the molecules under investigation, we have also performed XC-WF test calculations with extremely large λ_J_ values, actually showing that the electron densities computed with all the available diffraction data (sinθ/λ ≤ 2.0 Å^−1^) almost linearly approach the CCSD density (see Fig. S7 in the supporting information). However, even for λ_J_ = 100.0, which means that the structure factor fit accounts for 99% of functional (2) and the RHF part is just 1%, the distance from the CCSD density remains significant, as shown by the RSR indices (see Fig. S7).

All these results seem to indicate that, including only structure factor amplitudes up to a medium resolution of *ca* 0.7 Å^−1^, we capture completely the effects of the electron correlation on the valence component of the electron density. In contrast, the electron correlation effects on the core electron distribution are spread all over reciprocal space up to high-angle reflections. Their complete recovery might not be guaranteed, even using reflections up to a resolution higher than that considered in the present study.

For a more comprehensive comparison, we also discuss the performances of post-Hartree–Fock and DFT methods. The topological agreement index reveals that the CISD calculations retrieve a large part of the correlation effects introduced at the CCSD level (see Tables 1 ▸–3 ▸ ▸, and Tables S1–S13 in the supporting information). Of course, this is not surprising, given that many configurations are included in these calculations. At the same time, the computational cost of the CISD method remains very large. Concerning the DFT calculations, the results are quite heterogeneous and difficult to generalize, due to the less systematic nature of the density functionals. In fact, they sometimes provide ρ(**r**_b_) values that are even lower than those resulting from the coupled cluster calculations, but in other cases they perform worse than the CISD strategy. In view of this, one might anticipate that an X-ray constrained DFT strategy is not necessarily expected to work better than the original XC-RHF one. All the trends presented above agree with the analyses of the difference density maps (see Fig. 6 ▸, and Fig. S6 in the supporting information).

Finally, in Figs. 7 ▸ and 8 ▸ we show the detachment and attachment densities (Head-Gordon *et al.*, 1995 ▸) for the nitrogen and urea molecules (see Figs. S8–S11 in the supporting information for the detachment and attachment densities of the other investigated systems). As discussed in the previous section, in our study the detachment densities are defined as those parts of the unconstrained RHF electron distributions that are removed and rearranged into the correlated attachment electron densities (in our case, those associated with the CCSD, CISD and XC-RHF wave­functions). Here also, the attachment and detachment densities show that the main effect of the electron correlation is a shift in the charge distribution from the bonding region to the nuclei. This is clearly more pronounced at the CCSD and CISD levels. In fact, the surface isovalues used in Figs. 7 ▸ and 8 ▸ show that the CCSD and CISD methods provide larger electronic reorganizations of approximately the same order of magnitude. In contrast, for the XC-RHF wavefunctions the extent of the rearrangement is definitely smaller, especially when high-resolution structure factor amplitudes are included (sinθ/λ ≤ 1.5, 2.0 Å^−1^), in keeping with the results of the similarity indicators discussed above. Not surprisingly, the comparison with the CCSD attachment/detachment densities is better when only low/medium-angle reflections are taken into account (sinθ/λ ≤ 0.5, 0.7 Å^−1^). Nevertheless, the XC-HF approach always provides smaller reorganizations than the CCSD or CISD ones, which is further evidence of its limited ability to recover the entire effect of electron correlation. Therefore, the attachment and detachment densities also confirm the trends already observed in the difference density maps of the investigated systems (see Fig. 6 ▸, and Fig. S6 in the supporting information).

## 5. Conclusions   

We have carried out a thorough investigation of X-ray constrained wavefunction electron densities, with the aim of ascertaining whether the X-ray constrained wavefunction procedure is able to capture the effects of electron correlation on electron-density distributions. The study is based on the use of simulated scattering amplitudes computed for isolated molecules at a very high correlation level, in order to avoid perturbations due to crystal electric fields and/or biases due to experimental errors in the measurements. Under this hypothesis, an XC-WF should differ from an uncorrelated and unconstrained RHF wavefunction only by virtue of the electron correlation effects.

Within the framework of the XC-WF approach, a perfect density fit requires a very tight constraint (λ_J_ → ∞) and a scan of the entire reciprocal space (sinθ/λ → ∞). For practical reasons, neither condition can be fulfilled. In fact, convergence of the XC-WF with large λ_J_ is prohibitive and the resolution of measurable X-ray intensities is necessarily finite. Moreover, it is also important to note that simply fitting a wavefunction to a given electron density (which indeed corresponds to an ideal XC-WF procedure with λ_J_ → ∞ and sinθ/λ → ∞) would not guarantee finding the desired wavefunction (which was the original goal of the Jayatilaka approach), because, from a theoretical point of view, an infinite number of wavefunctions are actually compatible with a given electron distribution (Gilbert, 1975 ▸).

After analysing several molecules and using a realistically obtainable resolution of the X-ray data, we can conclude that the effect of electron correlation on electron density can be partially captured by the XC-WF method, provided that: (i) the external multiplier λ_J_ is sufficiently large and (ii) only low/medium-angle reflections are used as constraints in the functional to be minimized [see equations (2) and (3)]. In fact, on the one hand a large λ_J_ is necessary because weak effects, like those produced by electron correlation, require a very close fit, while on the other hand, low/medium-resolution data are crucial to recover completely the effect of the electron correlation on the valence component of the electron density. In contrast, to capture the subtler effects of the electron correlation on the core electron density, both a large value of λ_J_ and very high-resolution reflections might be necessary. A truncation of the structure factor resolution, even at the very high value of sinθ/λ = 2.0 Å^−1^, not only produces an incomplete fit of the core effects but, in the absence of an infinite λ_J_, it also drastically affects the capability of capturing the effects of electron correlation on the valence electron density, since the high-angle reflections become predominant in the functional to be minimized [see equations (2) and (3)]. As a consequence, a warning emerges from our analysis: the very small λ_J_ values and the high-resolution data typically adopted in XC-WF calculations with real experimental constraints may not guarantee a significant recovery of the electron correlation effects. Instead, it appears that a low-resolution fit is more efficient, retrieving at least the effects on the valence density and avoiding the counterproductive result of a partial fit of the correlation effects on the core electron density. Notably, it is plausible that electron distributions associated with frozen core correlated wavefunctions may be more easily fitted by XC-WF procedures with either medium or high-resolution data.

While the results presented in this paper clearly indicate a trend associated with XC-WFN analysis, the ‘critical’ values of λ_J_ and sinθ/λ cannot be generalized, because they depend on the radial distribution of each atom in the molecule. In fact, for complexes of transition metals, one may expect a larger critical resolution because of the more contracted nature of the metal *d* orbitals that obviously scatter to higher angles.

Further studies are still necessary to clarify better the physical meaning of the adjustable parameter λ_J_ and, consequently, the actual meaning of X-ray constrained wavefunctions obtained in cases in which the external (experimental or theoretical) constraint in equation (2) becomes largely predominant compared with the electronic energy of the system.

Furthermore, we envisage the need for other detailed studies where the effects of the crystal field (as distinct from electron correlation effects) can also be quantified and tested. In fact, as explained in the *Introduction*, the Hartree–Fock part of functional (2) is the Hamiltonian of an isolated unperturbed molecule, whereas the X-ray constrained part is generally linked to a periodic electron density. Moreover, the effect of real experimental data and experimental errors should be tested in more detail. In fact, it is very likely that the typical noise of experimental data may significantly affect the possibility of retrieving very small effects such as those due to electron correlation.

We believe that the results of this study and the follow up that we are planning could be of great importance, not only to define further the potentialities of the Jayatilaka approach, but also to propose new functionals within the framework of the density functional theory.

## Supplementary Material

Supporting information file. DOI: 10.1107/S2052252516019217/yc5009sup1.pdf

## Figures and Tables

**Table 1:** Electron density at the N—N bond critical point of the nitrogen molecule: topological agreement index associated with the correlated methods taken into account An index of 0.0 indicates perfect agreement with the CCSD density [see equation (8)].

	(sinθ/λ)_max_ (Å^−1^)					
Method	2.0	1.5	1.2	0.9	0.7	0.5
XC-RHF, λ_J_ = 0.5	99.76	99.41	98.84	97.37	95.12	100.62
XC-RHF, λ_J_ = 1.0	99.51	98.83	97.71	94.86	90.65	100.73
XC-RHF, λ_J_ = 1.5	99.27	98.25	96.60	92.46	86.52	100.45
XC-RHF, λ_J_ = 2.0	99.03	97.68	95.51	90.16	82.71	99.90
XC-RHF, λ_J_ = 2.5	98.79	97.12	94.44	87.97	79.17	99.18
XC-RHF, λ_J_ = 5.0	97.61	94.38	89.43	78.26	64.85	94.43
XC-RHF, λ_J_ = 7.5	96.44	91.78	84.87	70.31	54.46	89.46
XC-RHF, λ_J_ = 10.0	95.30	89.30	80.73	63.68	46.61	84.91
CISD	15.85					
BLYP	−1.81					
B3LYP	22.39					
VSXC	4.83					
B1B95	11.00					

**Table 2:** Electron density at the C—N bond critical point of the cyanide anion: topological agreement index associated with the correlated methods taken into account An index of 0.0 indicates perfect agreement with the CCSD density [see equation (8)].

	(sinθ/λ)_max_ (Å^−1^)					
Method	2.0	1.5	1.2	0.9	0.7	0.5
XC-RHF, λ_J_ = 0.5	99.77	99.42	98.82	97.21	94.59	97.10
XC-RHF, λ_J_ = 1.0	99.53	98.85	97.66	94.57	89.71	94.01
XC-RHF, λ_J_ = 1.5	99.30	98.28	96.54	92.08	85.29	91.00
XC-RHF, λ_J_ = 2.0	99.07	97.73	95.45	89.72	81.26	88.16
XC-RHF, λ_J_ = 2.5	98.84	97.18	94.38	87.48	77.59	85.52
XC-RHF, λ_J_ = 5.0	97.71	94.53	89.45	77.82	63.21	74.94
XC-RHF, λ_J_ = 7.5	96.60	92.04	85.07	70.14	53.23	67.40
XC-RHF, λ_J_ = 10.0	95.51	89.69	81.15	63.86	45.88	61.66
CISD	22.26					
BLYP	63.14					
B3LYP	83.45					
VSXC	−26.78					
B1B95	18.78					

**Table 3:** Electron density at the O—H bond critical point of the water molecule: topological agreement index associated with the correlated methods taken into account An index of 0.0 indicates perfect agreement with the CCSD density [see equation (8)].

	(sinθ/λ)_max_ (Å^−1^)					
Method	2.0	1.5	1.2	0.9	0.7	0.5
XC-RHF, λ_J_ = 0.5	99.72	99.34	98.76	97.38	95.17	93.63
XC-RHF, λ_J_ = 1.0	99.44	98.70	97.60	95.07	91.36	89.01
XC-RHF, λ_J_ = 1.5	99.17	98.08	96.50	93.02	88.24	85.29
XC-RHF, λ_J_ = 2.0	98.90	97.48	95.46	91.18	85.61	82.15
XC-RHF, λ_J_ = 2.5	98.63	96.90	94.47	89.52	83.33	79.39
XC-RHF, λ_J_ = 5.0	97.35	94.23	90.21	83.01	75.03	69.02
XC-RHF, λ_J_ = 7.5	96.14	91.89	86.81	78.34	69.31	61.91
XC-RHF, λ_J_ = 10.0	95.01	89.84	84.01	74.67	64.83	56.65
CISD	20.23					
BLYP	53.85					
B3LYP	54.33					
VSXC	123.27					
B1B95	53.53					
