# Supplementary material for: Can X-ray constrained Hartree–Fock wavefunctions retrieve electron correlation?
Source: IUCrJ. 2017 Jan 10;4(Pt 2):136–46. doi: 10.1107/S2052252516019217 (PMC5330524; doi:10.1107/S2052252516019217)
Supplement: Supplementary file 1 [file m-04-00136-sup1.pdf]

IUCrJ

Volume 4 (2017)

Supporting information for article:

Can X-ray constrained Hartree–Fock wavefunctions retrieve electron correlation?

Alessandro Genoni, Leonardo H. R. Dos Santos, Benjamin Meyer and Piero Macchi

Supporting Information available.

Tables S1-S13: values of the topological agreement index for the bond critical points of the urea, benzene and glycine molecules.

Figure S1: comparison between the CCSD electron densities and the RHF and XC-WF charge distributions along some selected chemical bonds of the six investigated molecules.

Figures S2-S4: values of the Carbó distances, the Root Mean Squared Deviations (RMSDs) and the Mean Absolute Deviations (MADs) between the CCSD electron densities and the XC-WF charge distributions in function of the external multiplier λ_J for all the examined molecules.

Figure S5: values of the topological agreement index associated with X-ray constrained wave functions (λ_J =10.0) for the different bond critical points of the molecules under exam in function of the resolution sinθ/λ.

Figure S6: representative isosurfaces of the difference density maps for the urea molecule using the CCSD charge distribution as reference.

Figure S7: values of the RSR similarity index until $\lambda_J = 100.0$ between the CCSD electron densities and the XC-WF charge distributions obtained from the complete set of reflections (sinθ/λ ≤ 2.0).

Figures S8-S11: representative isosurfaces of the detachment and attachment densities computed for the cyanide, water, benzene and glycine molecules.

Table S1 Electron density at the C1-O2 bond critical point of the urea molecule: topological agreement index associated with the correlated methods taken into account. An index of 0.0 indicates perfect agreement with the CCSD density (see equation (8) in the main text).

Method	$(sin\theta/\lambda)_{max}$ (Å^{-1})					
	2.0	1.5	1.2	0.9	0.7	0.5
XC-RHF / λ_J = 0.5	99.84	99.63	99.30	98.46	97.57	105.20
XC-RHF / λ_J = 1.0	99.68	99.27	98.61	96.95	95.23	108.71
XC-RHF / λ_J = 1.5	99.52	98.90	97.93	95.48	92.97	111.12
XC-RHF / λ_J = 2.0	99.36	98.54	97.26	94.04	90.80	112.79
XC-RHF / λ_J = 2.5	99.20	98.18	96.60	92.64	88.71	113.93
XC-RHF / λ_J = 5.0	98.42	96.42	93.40	86.15	79.44	115.55
XC-RHF / λ_J = 7.5	97.64	94.71	90.39	80.43	71.84	114.43
XC-RHF / λ_J = 10.0	96.88	93.05	87.56	75.36	65.56	112.57
CISD	35.06					
BLYP	-2.42					
B3LYP	25.00					
VSXC	-49.38					
B1B95	-20.94					

Table S2 Electron density at the C1-N3 bond critical point of the urea molecule: topological agreement index associated with the correlated methods taken into account. An index of 0.0 indicates perfect agreement with the CCSD density (see equation (8) in the main text).

Method	$(sin\theta/\lambda)_{max}$ (Å^{-1})					
	2.0	1.5	1.2	0.9	0.7	0.5
XC-RHF / λ_J = 0.5	99.86	99.66	99.35	98.51	97.04	98.74
XC-RHF / λ_J = 1.0	99.72	99.33	98.71	97.06	94.22	97.16
XC-RHF / λ_J = 1.5	99.57	99.00	98.07	95.65	91.54	95.46
XC-RHF / λ_J = 2.0	99.43	98.67	97.44	94.27	89.01	93.73
XC-RHF / λ_J = 2.5	99.29	98.34	96.81	92.92	86.60	92.04
XC-RHF / λ_J = 5.0	98.59	96.72	93.78	86.69	76.28	84.50
XC-RHF / λ_J = 7.5	97.90	95.14	90.91	81.20	68.12	78.57
XC-RHF / λ_J = 10.0	97.21	93.61	88.19	76.33	61.52	73.88
CISD	32.79					
BLYP	-52.86					
B3LYP	-19.32					
VSXC	-14.55					
B1B95	-34.23					

Table S3 Electron density at the N3-H4 bond critical point of the urea molecule: topological agreement index associated with the correlated methods taken into account. An index of 0.0 indicates perfect agreement with the CCSD density (see equation (8) in the main text).

Method	$(sin\theta/\lambda)_{max}$ (Å^{-1})					
	2.0	1.5	1.2	0.9	0.7	0.5
XC-RHF / λ_J = 0.5	99.82	99.56	99.14	98.01	95.95	92.29
XC-RHF / λ_J = 1.0	99.63	99.13	98.30	96.12	92.32	86.09
XC-RHF / λ_J = 1.5	99.45	98.70	97.49	94.33	89.04	80.90
XC-RHF / λ_J = 2.0	99.27	98.28	96.69	92.62	86.04	76.43
XC-RHF / λ_J = 2.5	99.09	97.86	95.90	90.99	83.27	72.51
XC-RHF / λ_J = 5.0	98.20	95.84	92.23	83.78	72.03	58.08
XC-RHF / λ_J = 7.5	97.33	93.92	88.89	77.77	63.62	48.55
XC-RHF / λ_J = 10.0	96.48	92.11	85.84	72.63	57.00	41.66
CISD	39.93					
BLYP	18.25					
B3LYP	30.49					
VSXC	47.90					
B1B95	24.63					

Table S4 Electron density at the N3-H5 bond critical point of the urea molecule: topological agreement index associated with the correlated methods taken into account. An index of 0.0 indicates perfect agreement with the CCSD density (see equation (8) in the main text).

Method	$(sin\theta/\lambda)_{max}$ (Å^{-1})					
	2.0	1.5	1.2	0.9	0.7	0.5
XC-RHF / λ_J = 0.5	99.80	99.52	99.07	97.83	95.58	91.72
XC-RHF / λ_J = 1.0	99.60	99.05	98.16	95.78	91.64	85.36
XC-RHF / λ_J = 1.5	99.40	98.58	97.27	93.83	88.10	80.25
XC-RHF / λ_J = 2.0	99.20	98.12	96.40	91.98	84.88	76.03
XC-RHF / λ_J = 2.5	99.00	97.67	95.55	90.22	81.95	72.45
XC-RHF / λ_J = 5.0	98.03	95.47	91.56	82.49	70.32	60.24
XC-RHF / λ_J = 7.5	97.08	93.39	87.96	76.17	62.03	52.86
XC-RHF / λ_J = 10.0	96.16	91.42	84.68	70.86	55.76	47.75
CISD	39.66					
BLYP	19.96					
B3LYP	31.01					
VSXC	54.53					
B1B95	24.41					

Table S5 Electron density at the C1-C2 bond critical point of the benzene molecule: topological agreement index associated with the correlated methods taken into account. An index of 0.0 indicates perfect agreement with the CCSD density (see equation (8) in the main text).

Method	$(sin\theta/\lambda)_{max}$ (Å^{-1})					
	2.0	1.5	1.2	0.9	0.7	0.5
XC-RHF / λ_J = 0.5	99.77	99.47	98.96	97.62	95.18	91.73
XC-RHF / λ_J = 1.0	99.55	98.94	97.95	95.36	90.84	85.05
XC-RHF / λ_J = 1.5	99.33	98.42	96.96	93.21	86.89	79.47
XC-RHF / λ_J = 2.0	99.11	97.91	96.00	91.16	83.29	74.70
XC-RHF / λ_J = 2.5	98.89	97.41	95.05	89.21	79.98	70.57
XC-RHF / λ_J = 5.0	97.81	94.95	90.59	80.60	66.80	56.01
XC-RHF / λ_J = 7.5	96.75	92.63	86.55	73.56	57.39	47.10
XC-RHF / λ_J = 10.0	95.71	90.42	82.86	67.66	50.33	41.04
CISD	37.68					
BLYP	-27.88					
B3LYP	-2.96					
VSXC	5.83					
B1B95	-17.63					

Table S6 Electron density at the C1-H7 bond critical point of the benzene molecule: topological agreement index associated with the correlated methods taken into account. An index of 0.0 indicates perfect agreement with the CCSD density (see equation (8) in the main text).

Method	$(sin\theta/\lambda)_{max}$ (Å^{-1})					
	2.0	1.5	1.2	0.9	0.7	0.5
XC-RHF / λ_J = 0.5	99.77	99.44	98.90	97.44	94.81	90.33
XC-RHF / λ_J = 1.0	99.53	98.88	97.82	95.00	90.10	82.06
XC-RHF / λ_J = 1.5	99.29	98.33	96.76	92.67	85.81	75.02
XC-RHF / λ_J = 2.0	99.06	97.78	95.72	90.45	81.90	68.98
XC-RHF / λ_J = 2.5	98.82	97.23	94.70	88.33	78.31	63.77
XC-RHF / λ_J = 5.0	97.66	94.60	89.90	79.03	64.15	45.72
XC-RHF / λ_J = 7.5	96.52	92.10	85.56	71.46	54.23	35.10
XC-RHF / λ_J = 10.0	95.41	89.72	81.60	65.18	46.91	28.14
CISD	48.66					
BLYP	-13.65					
B3LYP	11.16					
VSXC	18.29					
B1B95	5.71					

Table S7 Electron density at the N1-H2 bond critical point of the glycine molecule: topological agreement index associated with the correlated methods taken into account. An index of 0.0 indicates perfect agreement with the CCSD density (see equation (8) in the main text).

Method	$(sin\theta/\lambda)_{max}$ (Å^{-1})					
	2.0	1.5	1.2	0.9	0.7	0.5
XC-RHF / λ_J = 0.5	99.78	99.48	98.98	97.64	95.08	91.41
XC-RHF / λ_J = 1.0	99.56	98.96	97.98	95.39	90.62	84.21
XC-RHF / λ_J = 1.5	99.34	98.45	97.00	93.24	86.54	78.05
XC-RHF / λ_J = 2.0	99.12	97.94	96.05	91.18	82.79	72.68
XC-RHF / λ_J = 2.5	98.91	97.44	95.11	89.21	79.32	67.97
XC-RHF / λ_J = 5.0	97.83	95.01	90.67	80.43	65.27	50.91
XC-RHF / λ_J = 7.5	96.79	92.69	86.62	73.11	55.01	40.30
XC-RHF / λ_J = 10.0	95.76	90.49	82.90	66.90	47.21	33.13
CISD	44.90					
BLYP	13.03					
B3LYP	25.75					
VSXC	42.58					
B1B95	18.51					

Table S8 Electron density at the N1-C4 bond critical point of the glycine molecule: topological agreement index associated with the correlated methods taken into account. An index of 0.0 indicates perfect agreement with the CCSD density (see equation (8) in the main text).

Method	$(sin\theta/\lambda)_{max}$ (Å^{-1})					
	2.0	1.5	1.2	0.9	0.7	0.5
XC-RHF / λ_J = 0.5	99.89	99.73	99.48	98.84	97.61	96.87
XC-RHF / λ_J = 1.0	99.77	99.46	98.97	97.72	95.39	93.89
XC-RHF / λ_J = 1.5	99.66	99.20	98.47	96.64	93.30	91.08
XC-RHF / λ_J = 2.0	99.54	98.94	97.98	95.59	91.33	88.45
XC-RHF / λ_J = 2.5	99.43	98.68	97.49	94.58	89.46	85.98
XC-RHF / λ_J = 5.0	98.87	97.41	95.17	89.91	81.30	75.96
XC-RHF / λ_J = 7.5	98.32	96.18	92.99	85.77	74.65	68.79
XC-RHF / λ_J = 10.0	97.78	95.00	90.93	82.03	69.09	63.47
CISD	38.18					
BLYP	-42.86					
B3LYP	-13.83					
VSXC	5.70					
B1B95	-18.99					

Table S9 Electron density at the C4-H5 bond critical point of the glycine molecule: topological agreement index associated with the correlated methods taken into account. An index of 0.0 indicates perfect agreement with the CCSD density (see equation (8) in the main text).

Method	$(sin\theta/\lambda)_{max}$ (Å^{-1})					
	2.0	1.5	1.2	0.9	0.7	0.5
XC-RHF / λ_J = 0.5	99.85	99.65	99.31	98.39	96.68	94.54
XC-RHF / λ_J = 1.0	99.70	99.30	98.63	96.84	93.58	89.82
XC-RHF / λ_J = 1.5	99.55	98.95	97.96	95.33	90.67	85.69
XC-RHF / λ_J = 2.0	99.41	98.60	97.29	93.87	87.94	82.03
XC-RHF / λ_J = 2.5	99.26	98.26	96.64	92.44	85.36	78.74
XC-RHF / λ_J = 5.0	98.53	96.57	93.49	85.93	74.49	66.09
XC-RHF / λ_J = 7.5	97.81	94.94	90.52	80.27	66.10	57.26
XC-RHF / λ_J = 10.0	97.10	93.36	87.74	75.32	59.42	50.63
CISD	48.08					
BLYP	-18.57					
B3LYP	5.50					
VSXC	5.15					
B1B95	-2.73					

Table S10 Electron density at the C4-C7 bond critical point of the glycine molecule: topological agreement index associated with the correlated methods taken into account. An index of 0.0 indicates perfect agreement with the CCSD density (see equation (8) in the main text).

Method	$(sin\theta/\lambda)_{max}$ (Å^{-1})					
	2.0	1.5	1.2	0.9	0.7	0.5
XC-RHF / λ_J = 0.5	99.84	99.62	99.26	98.28	96.48	92.50
XC-RHF / λ_J = 1.0	99.68	99.25	98.53	96.63	93.24	86.39
XC-RHF / λ_J = 1.5	99.53	98.88	97.82	95.04	90.25	81.29
XC-RHF / λ_J = 2.0	99.37	98.51	97.12	93.52	87.47	76.94
XC-RHF / λ_J = 2.5	99.21	98.14	96.43	92.05	84.89	73.18
XC-RHF / λ_J = 5.0	98.44	96.36	93.14	85.46	74.25	59.87
XC-RHF / λ_J = 7.5	97.67	94.66	90.10	79.89	66.26	51.59
XC-RHF / λ_J = 10.0	96.93	93.02	87.29	75.09	60.01	45.85
CISD	39.18					
BLYP	-51.64					
B3LYP	-21.54					
VSXC	4.73					
B1B95	-24.26					

Table S11 Electron density at the C7-O8 bond critical point of the glycine molecule: topological agreement index associated with the correlated methods taken into account. An index of 0.0 indicates perfect agreement with the CCSD density (see equation (8) in the main text).

Method	$(sin\theta/\lambda)_{max}$ (Å^{-1})					
	2.0	1.5	1.2	0.9	0.7	0.5
XC-RHF / λ_J = 0.5	99.81	99.56	99.17	98.14	96.79	105.10
XC-RHF / λ_J = 1.0	99.62	99.13	98.36	96.35	93.78	108.13
XC-RHF / λ_J = 1.5	99.43	98.70	97.57	94.64	90.95	109.86
XC-RHF / λ_J = 2.0	99.24	98.28	96.80	92.99	88.28	110.76
XC-RHF / λ_J = 2.5	99.06	97.86	96.03	91.40	85.74	111.10
XC-RHF / λ_J = 5.0	98.13	95.83	92.43	84.22	74.87	109.16
XC-RHF / λ_J = 7.5	97.23	93.90	89.13	78.10	66.29	105.48
XC-RHF / λ_J = 10.0	96.35	92.05	86.08	72.78	59.37	101.78
CISD	42.61					
BLYP	14.76					
B3LYP	39.77					
VSXC	-46.52					
B1B95	-13.55					

Table S12 Electron density at the C7-O9 bond critical point of the glycine molecule: topological agreement index associated with the correlated methods taken into account. An index of 0.0 indicates perfect agreement with the CCSD density (see equation (8) in the main text).

Method	$(sin\theta/\lambda)_{max}$ (Å^{-1})					
	2.0	1.5	1.2	0.9	0.7	0.5
XC-RHF / λ_J = 0.5	99.72	99.56	99.39	99.74	102.18	158.53
XC-RHF / λ_J = 1.0	99.55	99.24	98.93	99.69	104.29	202.31
XC-RHF / λ_J = 1.5	99.38	98.94	98.50	99.69	106.10	235.41
XC-RHF / λ_J = 2.0	99.21	98.64	98.11	99.73	107.55	260.73
XC-RHF / λ_J = 2.5	99.05	98.36	97.74	99.79	108.67	280.27
XC-RHF / λ_J = 5.0	98.27	97.07	96.21	100.04	109.97	329.27
XC-RHF / λ_J = 7.5	97.55	95.97	95.07	99.82	106.61	341.71
XC-RHF / λ_J = 10.0	96.87	95.03	94.16	99.02	101.00	341.19
CISD	50.71					
BLYP	-36.84					
B3LYP	44.28					
VSXC	-254.03					
B1B95	-190.55					

Table S13 Electron density at the O9-H10 bond critical point of the glycine molecule: topological agreement index associated with the correlated methods taken into account. An index of 0.0 indicates perfect agreement with the CCSD density (see equation (8) in the main text).

Method	$(sin\theta/\lambda)_{max}$ (Å^{-1})					
	2.0	1.5	1.2	0.9	0.7	0.5
XC-RHF / λ_J = 0.5	99.51	98.86	97.82	95.10	90.02	84.33
XC-RHF / λ_J = 1.0	99.03	97.75	95.74	90.68	81.85	73.65
XC-RHF / λ_J = 1.5	98.55	96.66	93.74	86.66	75.02	65.88
XC-RHF / λ_J = 2.0	98.07	95.59	91.83	83.00	69.20	59.98
XC-RHF / λ_J = 2.5	97.60	94.55	90.00	79.63	64.18	55.34
XC-RHF / λ_J = 5.0	95.32	89.68	81.84	66.21	46.55	41.93
XC-RHF / λ_J = 7.5	93.15	85.30	75.04	56.57	35.77	35.52
XC-RHF / λ_J = 10.0	91.07	81.33	69.28	49.24	28.40	31.73
CISD	49.22					
BLYP	89.70					
B3LYP	77.00					
VSXC	163.36					
B1B95	61.22					

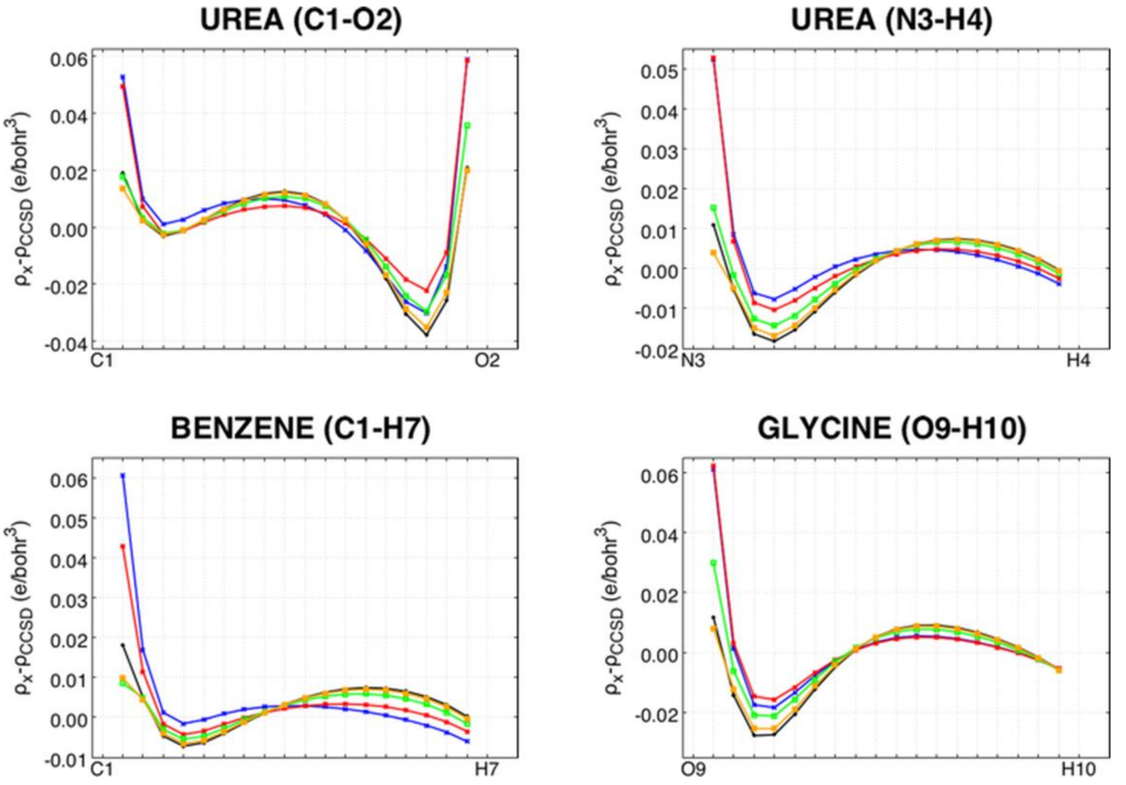

Figure S1 Comparison between the CCSD electron densities and the RHF (black), XC-WF/0.5 (blue), XC-WF/0.7 (red), XC-WF/1.2 (green) and XC-WF/2.0 (orange) charge distributions along some selected chemical bonds of the six investigated molecules.

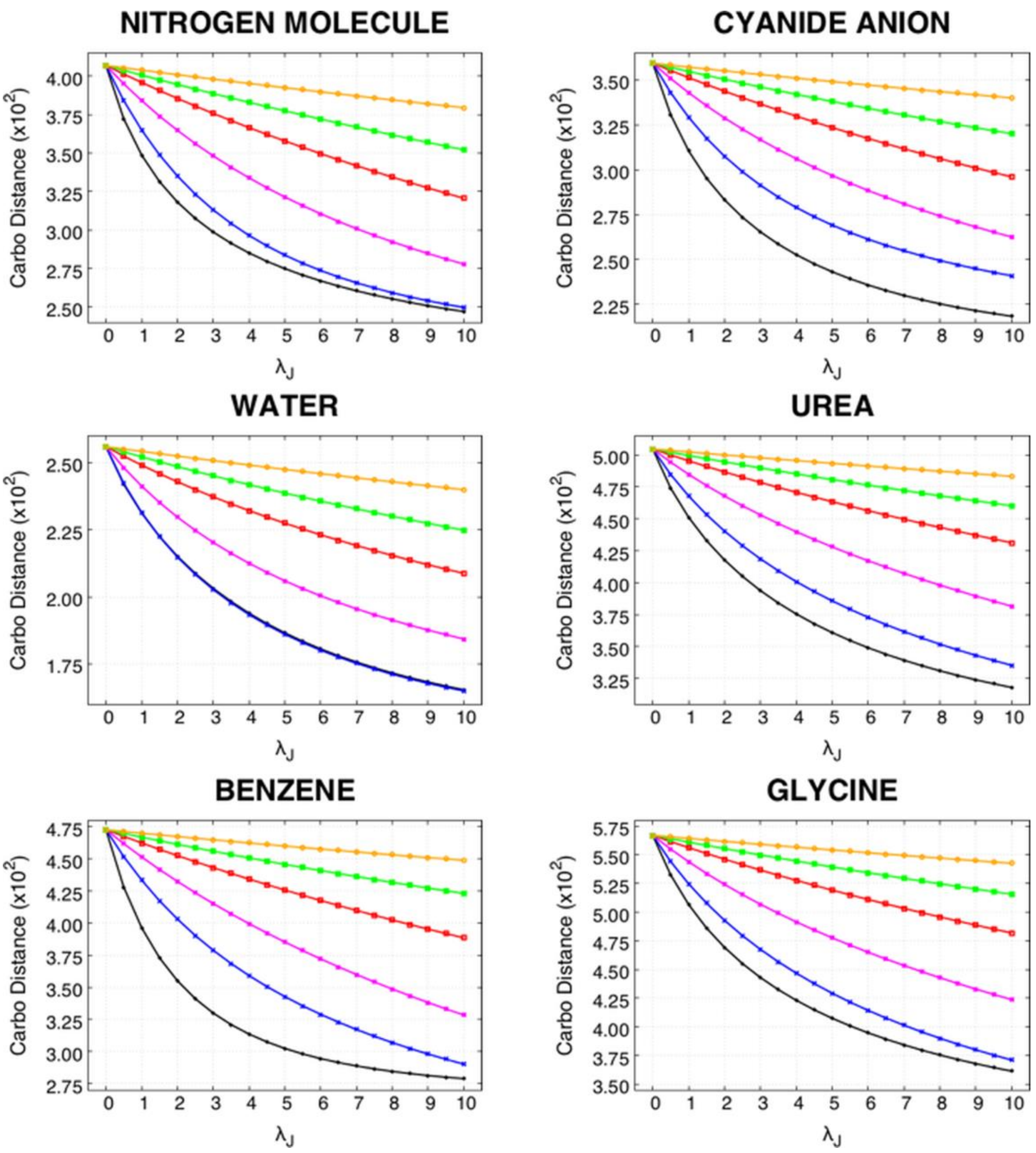

Figure S2 Values of the Carbó distances between the CCSD electron densities and the XC-WF charge distributions in function of the external multiplier λ_J for all the examined molecules. The yellow, green, red, magenta, blue and black curves correspond to XC-WF calculations performed with structure factor amplitudes up to a resolution of 2.0, 1.5, 1.2, 0.9, 0.7 and 0.5 Å^{-1}, respectively.

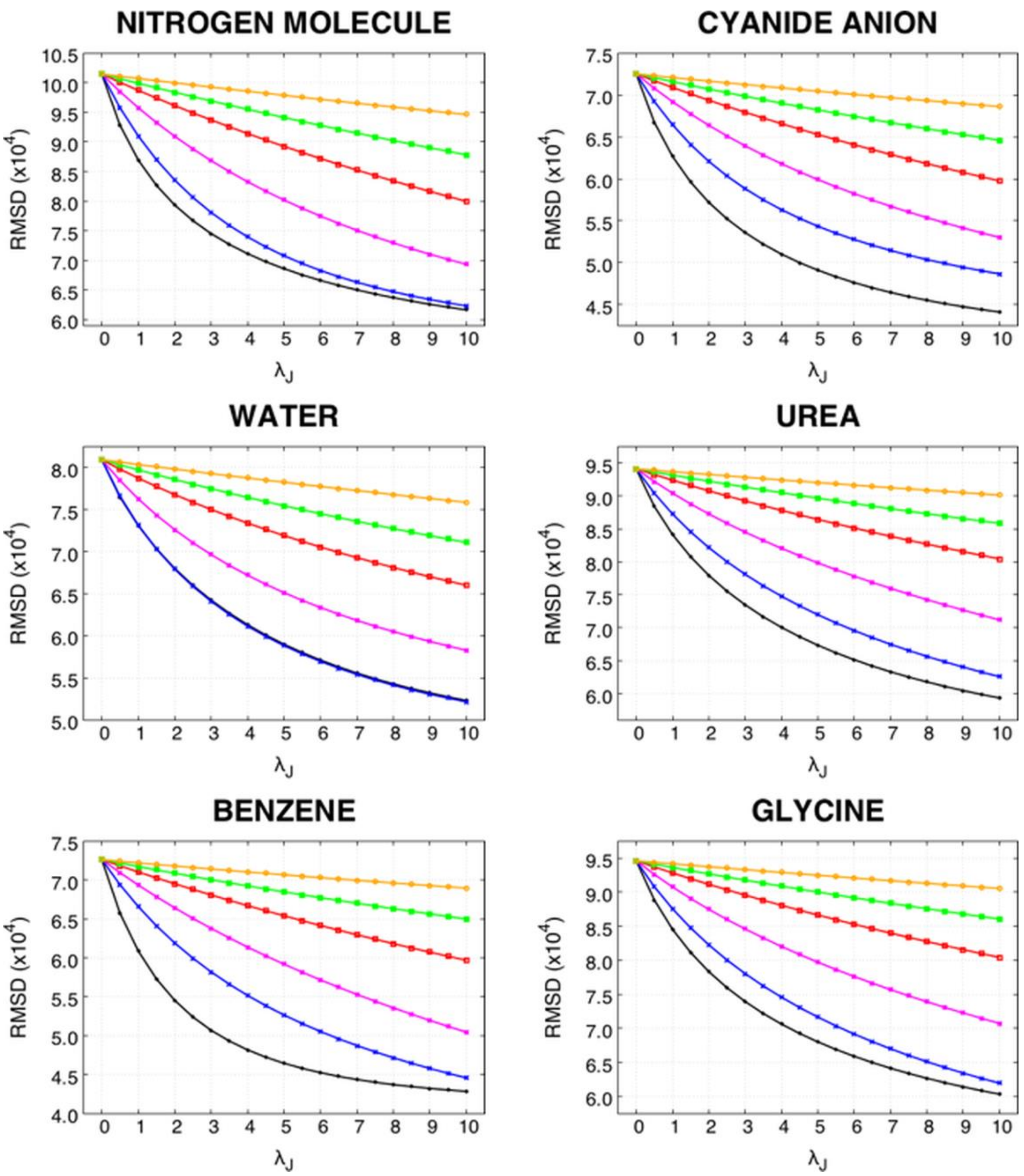

Figure S3 Values of the Root Mean Square Deviations between the CCSD electron densities and the XC-WF charge distributions in function of the external multiplier λ_J for all the examined molecules. The yellow, green, red, magenta, blue and black curves correspond to XC-WF calculations performed with structure factor amplitudes up to a resolution of 2.0, 1.5, 1.2, 0.9, 0.7 and 0.5 Å^{-1}, respectively.

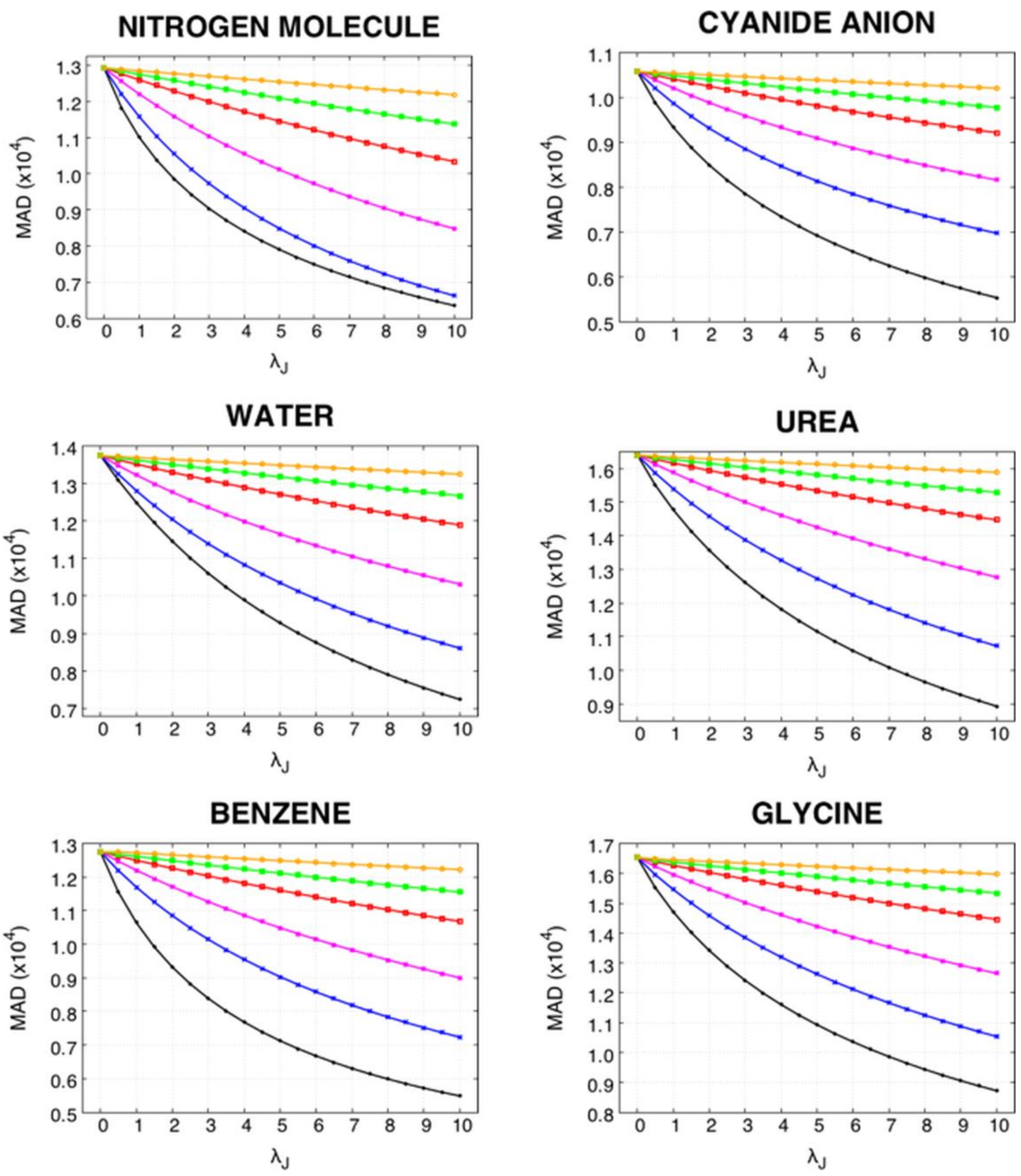

Figure S4 Values of the Mean Absolute Deviations between the CCSD electron densities and the XC-WF charge distributions in function of the external multiplier λ_J for all the examined molecules. The yellow, green, red, magenta, blue and black curves correspond to XC-WF calculations performed with structure factor amplitudes up to a resolution of 2.0, 1.5, 1.2, 0.9, 0.7 and 0.5 Å^{-1}, respectively.

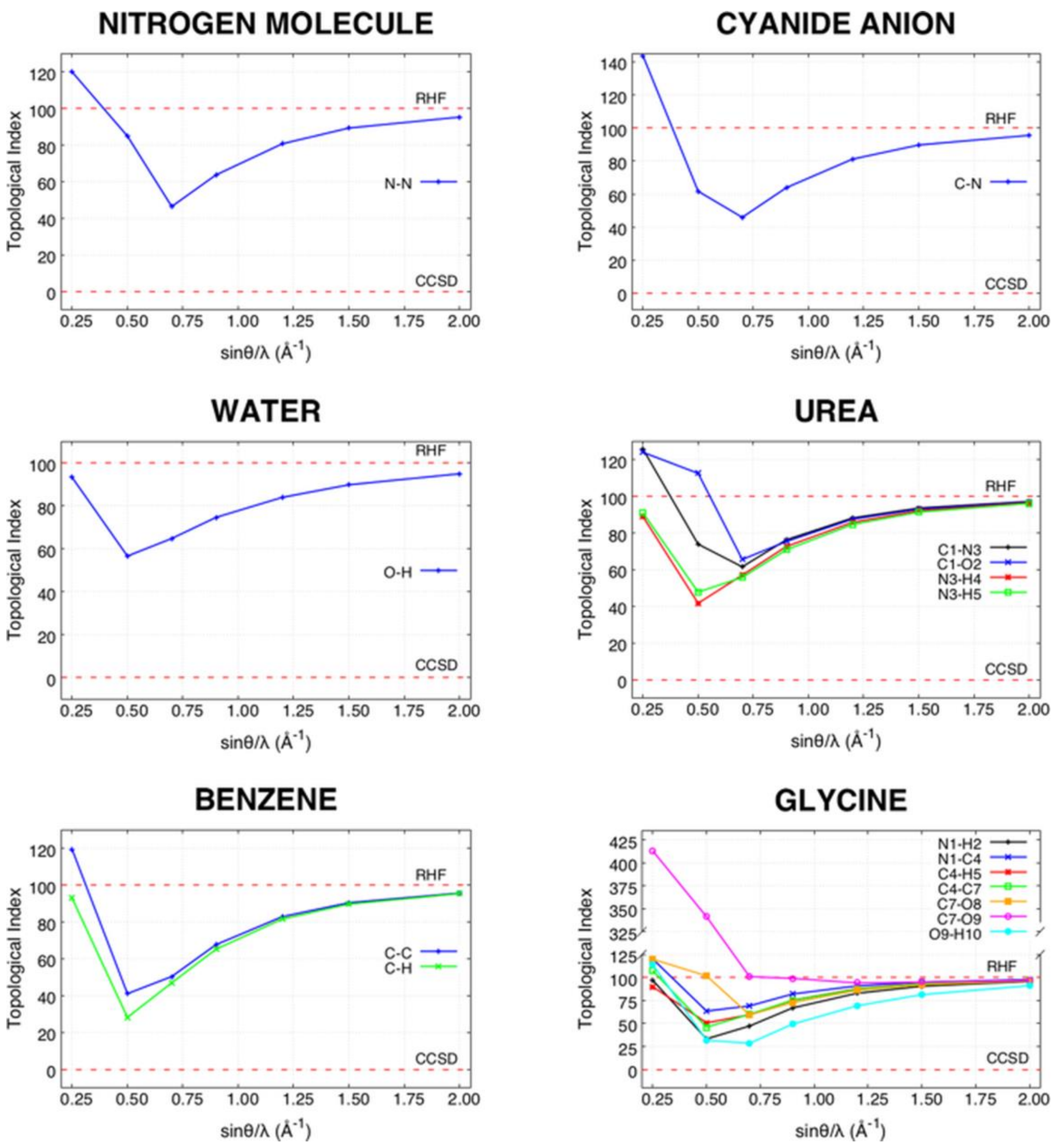

Figure S5 Values of the topological agreement index associated with X-ray constrained wave functions (λ_J = 10.0) for the different bond critical points of the molecules under exam in function of the resolution sinθ/λ. The two red dashed-lines represent the RHF and CCSD benchmarks.

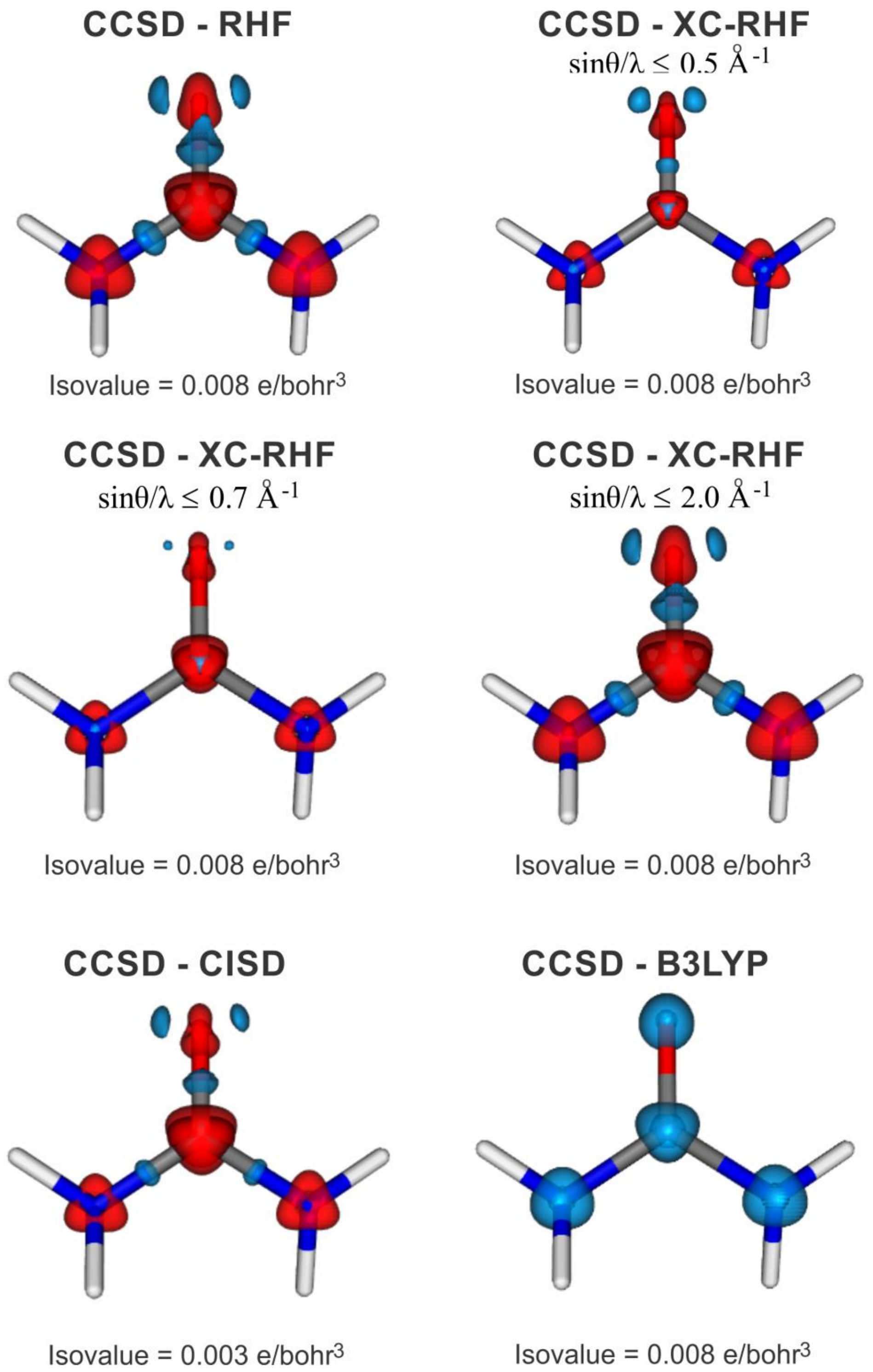

Figure S6 Representative isosurfaces of the difference density maps for the urea molecule using the CCSD charge distribution as reference. The chosen isovalue is always 0.008 e/bohr^3, except for the CCSD-CISD difference density, for which the isovalue has been set equal to 0.003 e/bohr^3. Positive and negative isosurfaces are depicted in red and blue, respectively.

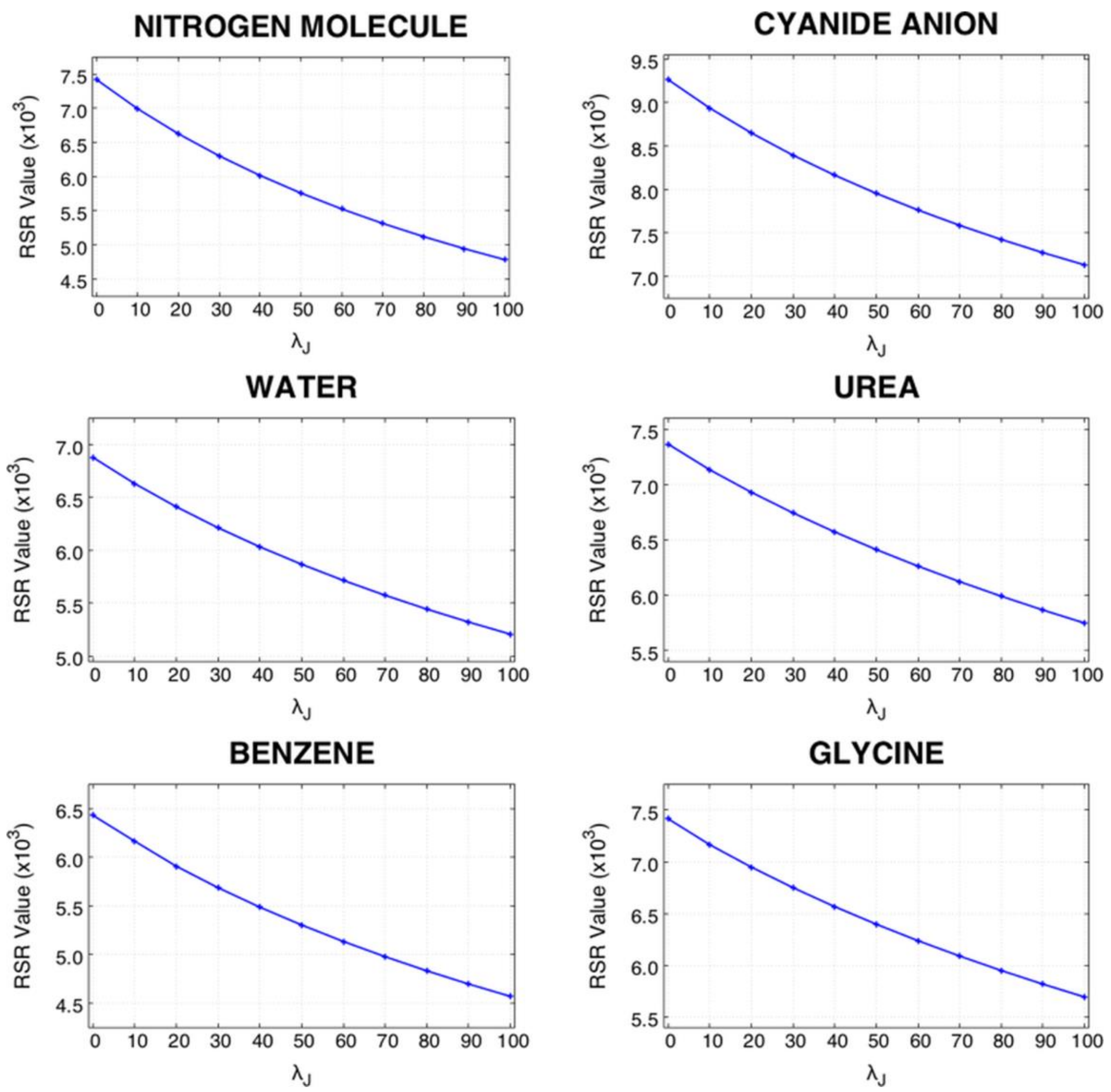

Figure S7 Values of the RSR similarity indexes (until $\lambda_J = 100.0$) between the CCSD electron densities and the XC-WF charge distributions obtained from the complete set of reflections ($\sin\theta/\lambda \leq 2.0$).

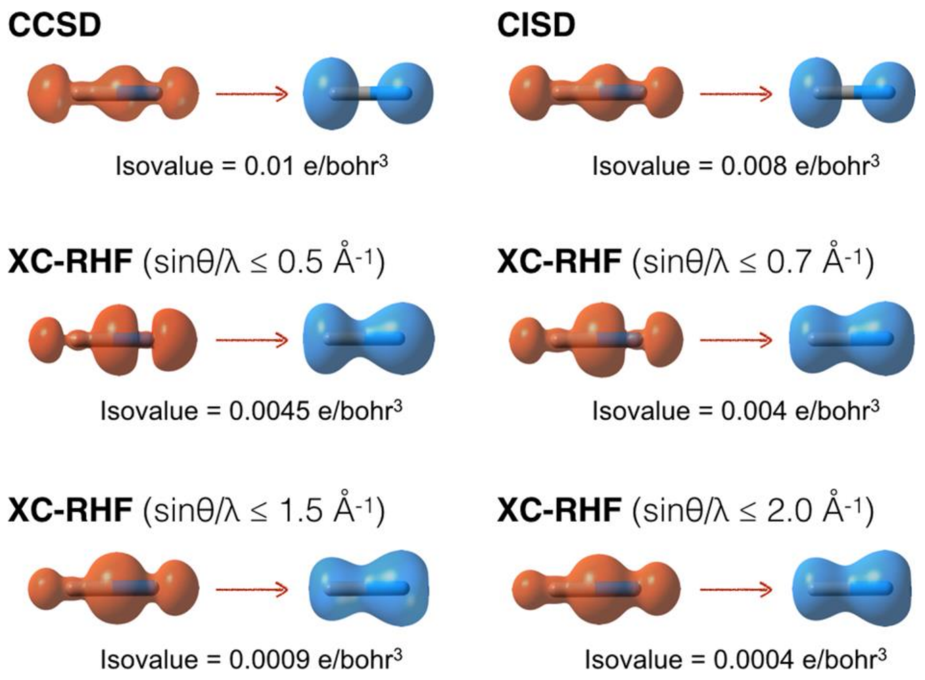

Figure S8 Representative isosurfaces of the detachment and attachment densities (in orange and blue, respectively) of the cyanide anion relative to electronic rearrangements with respect to the reference RHF charge distribution when different correlated wave functions are taken into account.

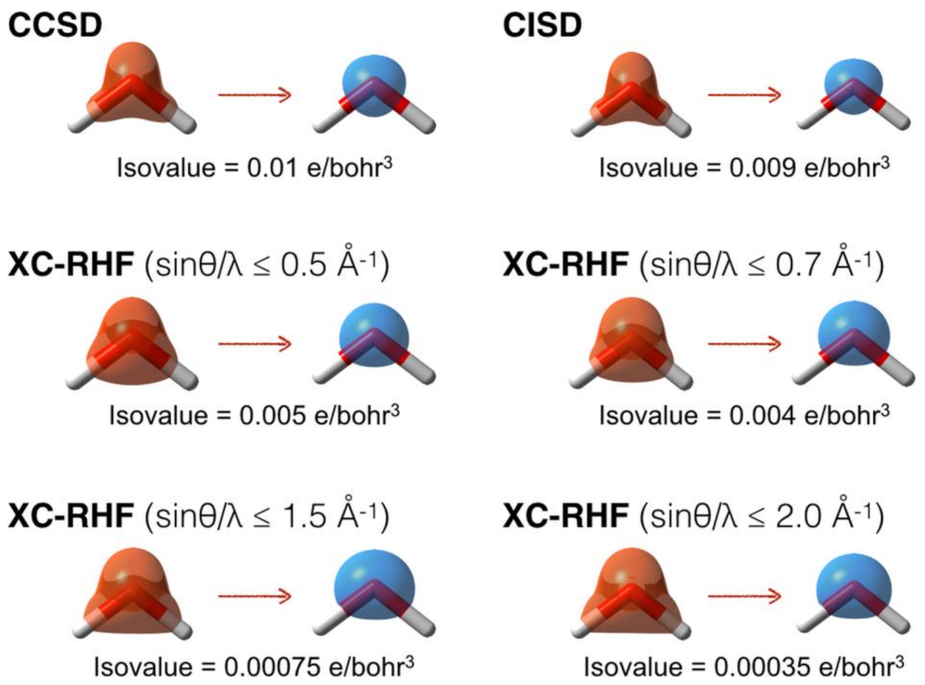

Figure S9 Representative isosurfaces of the detachment and attachment densities (in orange and blue, respectively) of the water molecule relative to electronic rearrangements with respect to the reference RHF charge distribution when different correlated wave functions are taken into account.

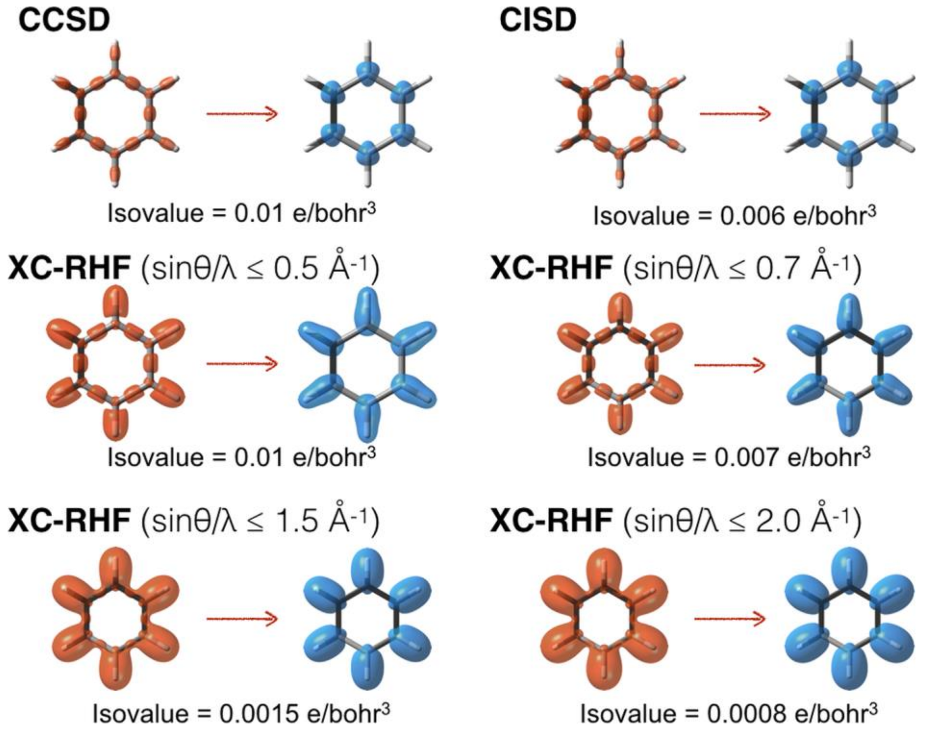

Figure S10 Representative isosurfaces of the detachment and attachment densities (in orange and blue, respectively) of the benzene molecule relative to electronic rearrangements with respect to the reference RHF charge distribution when different correlated wave functions are taken into account.

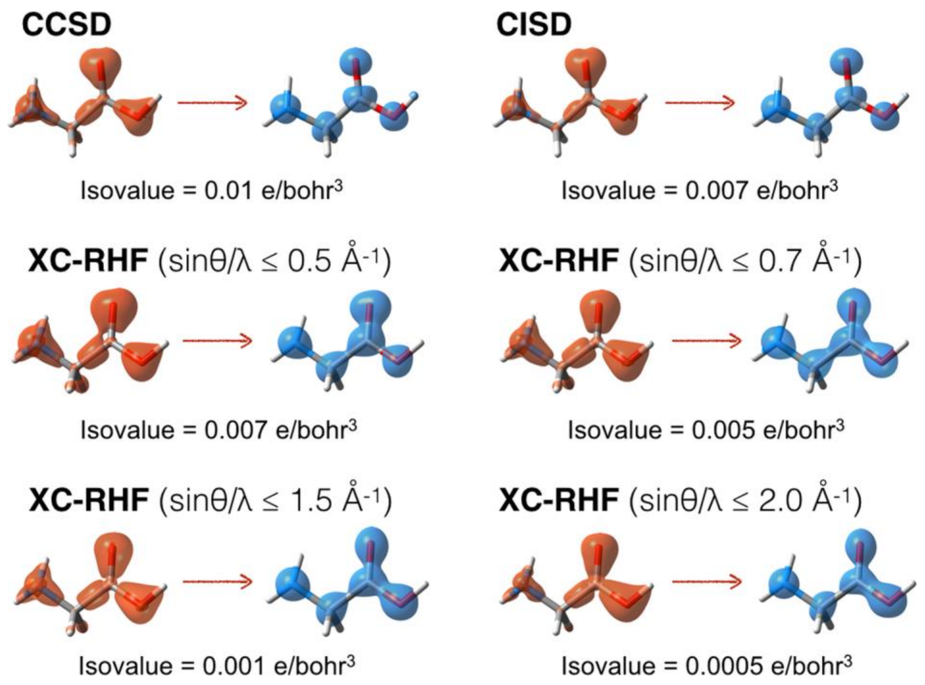

Figure S11 Representative isosurfaces of the detachment and attachment densities (in orange and blue, respectively) of the glycine molecule relative to electronic rearrangements with respect to the reference RHF charge distribution when different correlated wave functions are taken into account.
